# Bempegaldesleukin selectively depletes intratumoral Tregs and potentiates T cell-mediated cancer therapy

**DOI:** 10.1038/s41467-020-14471-1

**Published:** 2020-01-31

**Authors:** Meenu Sharma, Hiep Khong, Faisal Fa’ak, Salah-Eddine Bentebibel, Louise M. E. Janssen, Brent C. Chesson, Caitlin A. Creasy, Marie-Andrée Forget, Laura Maria S. Kahn, Barbara Pazdrak, Binisha Karki, Yared Hailemichael, Manisha Singh, Christina Vianden, Srinivas Vennam, Uddalak Bharadwaj, David J. Tweardy, Cara Haymaker, Chantale Bernatchez, Shixia Huang, Kimal Rajapakshe, Cristian Coarfa, Michael E. Hurwitz, Mario Sznol, Patrick Hwu, Ute Hoch, Murali Addepalli, Deborah H. Charych, Jonathan Zalevsky, Adi Diab, Willem W. Overwijk

**Affiliations:** 1 Department of Melanoma Medical Oncology, University of Texas MD Anderson Cancer Center, Houston, TX USA; 2 Nektar Therapeutics, 455 Mission Bay Blvd South, San Francisco, CA USA; 3 Department of Infectious Diseases, Infection Control and Employee Health, The University of Texas MD Anderson Cancer Center, Houston, TX 77054 USA; 4 Department of Molecular and Cellular Biology, Baylor College of Medicine, Houston, TX USA; 5 Dan L. Duncan Cancer Center, Houston, TX USA; 6 Yale Comprehensive Cancer Center, New Haven, CT USA; 7 Yale University Cancer Center, Yale University, New Haven, CT USA; 8 The University of Texas MD Anderson Cancer Center UT Health Graduate School of Biomedical Sciences, Houston, TX USA; 9 Department of Immunology, University of Texas MD Anderson Cancer Center, Houston, TX USA

**Keywords:** Cancer therapy, Tumour immunology, Cytokines, Immunotherapy, Lymphocytes

## Abstract

High dose interleukin-2 (IL-2) is active against metastatic melanoma and renal cell carcinoma, but treatment-associated toxicity and expansion of suppressive regulatory T cells (Tregs) limit its use in patients with cancer. Bempegaldesleukin (NKTR-214) is an engineered IL-2 cytokine prodrug that provides sustained activation of the IL-2 pathway with a bias to the IL-2 receptor CD122 (IL-2Rβ). Here we assess the therapeutic impact and mechanism of action of NKTR-214 in combination with anti-PD-1 and anti-CTLA-4 checkpoint blockade therapy or peptide-based vaccination in mice. NKTR-214 shows superior anti-tumor activity over native IL-2 and systemically expands anti-tumor CD8^+^ T cells while inducing Treg depletion in tumor tissue but not in the periphery. Similar trends of intratumoral Treg dynamics are observed in a small cohort of patients treated with NKTR-214. Mechanistically, intratumoral Treg depletion is mediated by CD8^+^ Teff-associated cytokines IFN-γ and TNF-α. These findings demonstrate that NKTR-214 synergizes with T cell-mediated anti-cancer therapies.

## Abstract (web summary)

Interleukin-2 can induce an anti-tumour response, but is associated with toxicity. Here, the authors demonstrate that an engineered interleukin-2 promotes intratumoral T regulatory cell depletion while enhancing effective anti-tumour CD8^+^ T cell responses that result in potent tumor suppression.

## Introduction

Therapies that harness the immune system to fight cancer are continuing to improve outcomes for patients with cancer. Checkpoint inhibitor (CPI) therapy is based on blocking engagement of the inhibitory CTLA-4 and PD-1 receptors on tumor-specific Teff, with the primary effect of invigorating their antitumor function in the immunosuppressive tumor microenvironment^1,2^. Other immune therapies such as vaccination increase the priming of naive T cells into Teff, while cytokine-based therapies seek to increase the proliferation and survival of pre-existing Teff. One FDA-approved cytokine-based therapy, high-dose IL-2 therapy (HD IL-2), yields a 15–20% objective response rate in metastatic renal cell carcinoma and melanoma^3,4^, and a further improved clinical response was observed when HD IL-2 was combined with a melanoma peptide vaccine^5,6^. However, vascular leak syndrome, hypotension, and liver toxicities associated with HD IL-2 have limited its use in cancer immunotherapy. In addition, HD IL-2 can expand potently suppressive CD4^+^CD25^+^ Foxp3^+^ Tregs in cancer patients, possibly limiting its efficacy^7–10^.

IL-2 stimulates T-cell growth and proliferation by binding through the high-affinity trimeric IL-2 receptor (IL-2R) consisting of alpha (CD25), beta (CD122), and common cytokine-receptor gamma (CD132) chain. Naive CD8^+^ T cells express low-affinity dimeric IL-2R (CD122/132) and transiently express CD25 after TCR stimulation^11^. In contrast, Tregs constitutively express high levels of CD25^11^, allowing them to successfully compete for IL-2, which has a very short half-life of ~7 min in humans^12^. CD25 is also expressed on endothelial cells and has been implicated in some of IL-2’s toxic vascular effects^13^. Together, the biology of IL-2 and its receptor expression suggests that IL-2 analogs that are more long-lived and preferentially target the CD122/CD132 intermediate-affinity receptor over the CD122/CD132/CD25 high-affinity receptor will preferentially support Teff over Tregs and will cause less toxicity. In addition, such an IL-2 analog could be expected to synergize with anti-CTLA-4 and anti-PD-1 CPI therapy and/or vaccination through numerical expansion and further activation of tumor-specific Teff subsets.

NKTR-214 is an engineered IL-2R agonist with an average of six releasable polyethylene glycol (PEG) molecules attached to the IL-2Rα binding region of IL-2 (aldesleukin). This site-specific PEGylation preferentially reduces IL-2 binding to CD25 over CD122/CD132^14,15^. In early preclinical studies, NKTR-214 monotherapy induced tumor regression accompanied by preferential intratumoral expansion of Teff over Tregs in various immunogenic murine tumor models^14^. In patients with metastatic melanoma and renal cell cancer, including those who had failed PD-1 CPI therapy, NKTR-214 monotherapy enhanced peripheral and intratumoral CD8^+^ T-cell proliferation and numbers without increasing intratumoral Tregs, and without causing serious toxicity^16^. To understand the activity and mechanism of action of NKTR-214 in T-cell-based immunotherapy of cancer, we studied its use in preclinical models of PD-1 and CTLA-4 CPI therapy and in antigen-specific vaccination as well as in patient samples. Here, we report that NKTR-214 promotes tumor regression in a variety of animal models of CPI therapy and vaccination. NKTR-214 selectively expands intratumoral Teff over Tregs, and induces Teff-derived cytokine release that drives selective depletion of Tregs in the tumor tissue but not the periphery. Intratumoral Treg depletion was not only limited to animal models, but also observed in patients treated with NKTR-214. Together, these results shed light on the mechanism of action of NKTR-214 and inform its clinical application in combination with immune-based therapies for patients with cancer.

## Results

### NKTR-214 synergizes with checkpoint blockade therapy

**Fig. 1:**
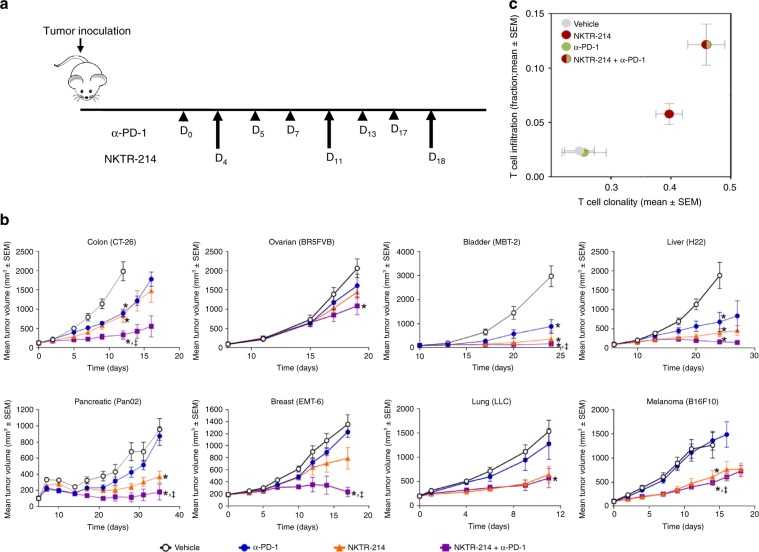
NKTR-214 synergizes with checkpoint blockade therapy. **a**–**c** Mice bearing s.c. tumors LLC, Pan02, B16.F10 (in C57BL/6 strain), MBT-2 (C3H), CT26, H22, EMT6 (BALB/c), and BR5FVB (FVB) were treated with vehicle or received anti-PD-1 (α-PD-1) and/or NKTR-214 as indicated. **a** Experimental scheme. **b** Tumor size. *p < 0.05 compared with vehicle, ^‡^*p* < 0.05 compared with single agent anti-PD-1, and unpaired *t* test at the day of study end, defined as the day when ~20% vehicle group was euthanized for tumor burden. **c** T-cell clonality and infiltration were assessed in CT26 tumors 7 days after the indicated treatment was initiated. TCR Vβ and Jβ usage was determined by utilizing the ImmunoSEQ platform from Adaptive Biotechnologies. The results are the average of four replicates per cohort.

To explore whether NKTR-214 could improve T-cell-mediated antitumor immunity induced by PD-1 CPI therapy, we evaluated the efficacy of anti-PD-1 or NKTR-214 alone and in combination, in eight different tumor models of colon, ovarian, bladder, liver, pancreatic, breast and lung cancer, and melanoma in the four different mouse strains BALB/c, C57BL/6, C3H, and FVB. We found superior activity of the combination over anti-PD-1 monotherapy in all models tested (Fig. 1a, b). In five out of eight models, the combination was also more potent than NKTR-214 monotherapy, indicating benefit of the combination over either monotherapy in the majority of models tested. Similarly, the combination of NKTR-214 with anti-PD-1 more potently suppressed rapidly growing CT26 tumors than aldesleukin with anti-PD-1 (Supplementary Fig. 1a). No dose-limiting toxicity was observed in any of the tumor models or mouse strains. We also explored whether NKTR-214 could potentiate dual PD-1/CTLA-4 CPI therapy in a more stringent therapeutic model of advanced CT26 tumors. In this setting, none of the monotherapies were effective, while a combination of anti-PD-1 and anti-CTLA-4 cured 44% of the animals; addition of NKTR-214 increased this to 66% cure (Supplementary Fig. 1b, c). Together, these results indicate therapeutic benefit of NKTR-214 in combination with CPI therapy in multiple mouse strains and tumor models.

To gain insight into immune mechanisms underlying the antitumor activity of NKTR-214, we more closely examined therapy-induced tumor-infiltrating T cells (TILs). A recent report demonstrated that only ~10% of TILs in human tumors are truly tumor-reactive, suggesting that only some TILs contribute actively to tumor control^17^. One measure of the antitumor activity of TILs is their clonality, as defined by TCR Vβ and Jβ usage. Indeed, the presence of high-frequency (and presumably tumor antigen-reactive) T-cell clones correlated with improved response in patients with prostate cancer treated with anti-CTLA-4 and a cancer vaccine^18^. Using T-cell DNA quantification and T-cell receptor sequencing, we found that on day 7 post treatment when tumor size had begun to decrease, anti-PD-1 monotherapy did not change TIL frequency or clonality in the CT26 tumor model, while NKTR-214 increased both TIL frequency and clonality as a monotherapy and more potently when combined with anti-PD-1 (Fig. 1c). Using fingolimod (which sequesters lymphocytes in lymph nodes), we found that NKTR-214 increased the intratumoral accumulation of CD8^+^ T cells even as their numbers in the circulation were reduced due to inhibited egress from lymph nodes (Supplementary Fig. 1d). Relative to NKTR-214 alone, NKTR-214 and anti-PD-1 combination resulted in somewhat lower levels of CD8^+^ T cells in the blood, which could be due to enhanced transitioning of CD8^+^ T cells from the blood into the tumor mass as observed in this combination treatment group (Supplementary Fig. 1d). Together, these results demonstrate that NKTR-214 potentiates antitumor T cells and tumor regression after anti-PD-1 CPI therapy.

### NKTR-214 expands and maintains vaccination-induced Teff

To develop a more in-depth understanding of the immunological mechanism of action of NKTR-214, we selected a tumor model that allowed for detailed analysis of tumor-specific T-cell responses, hence the pmel-1/B16.F10 melanoma model that employs trackable, CD90.1 congenically marked gp100 melanoma antigen-specific CD8^+^ T cells was used^19–21^.

**Fig. 2:**
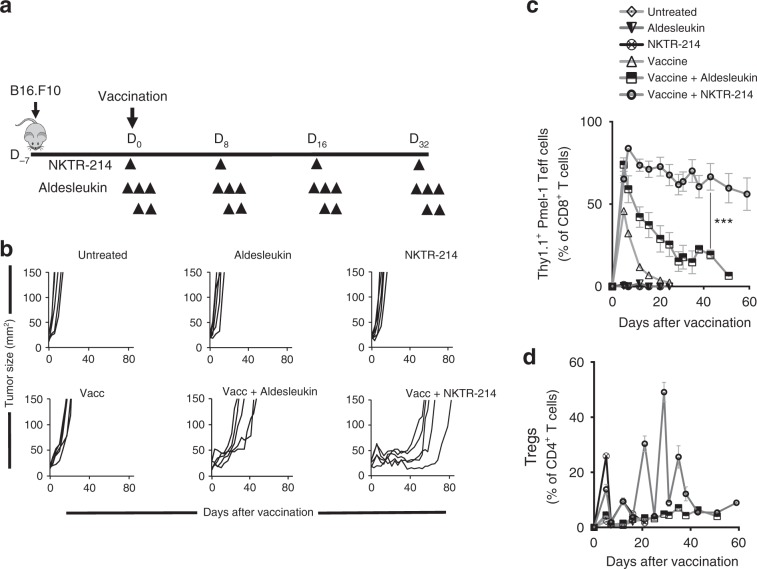
NKTR-214 supports vaccination-induced, antitumor Teff. **a**–**d** C57BL/6 mice bearing 7-day-old, s.c. B16.F10 tumors received pmel-1 T-cell and gp100 peptide vaccination followed by either aldesleukin or NKTR-214. **a** Experimental scheme. **b** Tumor size in individual mice. **c** Pmel-1 CD8^+^ Teff, and **d** CD4^+^ CD25^hi^ Foxp3^+^ Tregs in blood through time. Data are represented as mean ± SEM (*n* = 5, **P < 0.01, unpaired *t* test).

We treated B16.F10 tumor-bearing mice with gp100 peptide vaccination alone or in combination with either five doses of aldesleukin (the standard regimen^19^, once on day 0 and twice on days 1 and 2) or a single dose of NKTR-214 (Fig. 2a). This dosing of aldesleukin and NKTR-214 was repeated every 8 days. NKTR-214 and aldesleukin monotherapy did not suppress tumor growth (Fig. 2b). A combination of vaccination and NKTR-214 markedly suppressed tumor growth and prolonged mouse survival for up to 2 months (Fig. 2b and Supplementary Fig. 2d).

Long-term tumor control after vaccine plus NKTR-214 directly correlated with the magnitude and persistence of vaccination-induced, gp100-specific CD8^+^ Teff in the circulation (Fig. 2c). Aldesleukin also synergized with vaccination, but despite a variety of schedules and dose ranges tested, it never approached the potency of NKTR-214 given every 8 days in terms of T-cell response or tumor control (Supplementary Fig. 2a, b, d). NKTR-214 also drove the expansion of CD4^+^Foxp3^+^ Tregs in the circulation; however, this expansion was transient and followed by rapid contraction toward the end of each 8-day cycle of NKTR-214 dosing, resulting in a cyclical pattern that was in contrast with the stable enhancement of CD8^+^ Teff responses in the same animals (Fig. 2c, d). It is likely that due to its short in vivo half-life, the effect of aldesleukin on peripheral Tregs may not be accurately captured at these time points in the analysis. All mice eventually succumbed to progressively growing tumors. Similar to the findings by Landsberg et al. in the HGF-CDK4 melanoma model, we found that B16.F10 tumors also develop resistance to gp100-specific CD8^+^ T cells due to downregulation of gp100 antigen expression caused by T-cell-derived cytokines^22^. We confirmed that recurring B16.F10 tumors had lost gp100 antigen expression (Supplementary Fig. 2e), which coincided with a reduction of CD8^+^ Teff numbers and a concomitant rise in intratumoral Tregs (Supplementary Fig. 2f). These data demonstrate the strong antigen-specific selection pressure on tumor cells by gp100-specific CD8^+^ Teff after gp100 vaccination and NKTR-214, and support the notion that anticancer vaccines for patients with cancer will ideally target multiple tumor-associated antigens to minimize tumor escape through single antigen loss. We further validated the antitumor efficacy of NKTR-214 in a AH-1 antigenic peptide vaccine model of large CT26 tumors^23^, where we observe enhanced tumor control of single-dose peptide vaccine plus NKTR-214 compared with vaccine or NKTR-214 alone (Supplementary Fig. 3a–c). Together, these results indicate that NKTR-214 enhances CD8^+^ Teff responses and tumor control after anticancer vaccination.

### NKTR-214 promotes Teff expansion and depletes Tregs in tumor

**Fig. 3:**
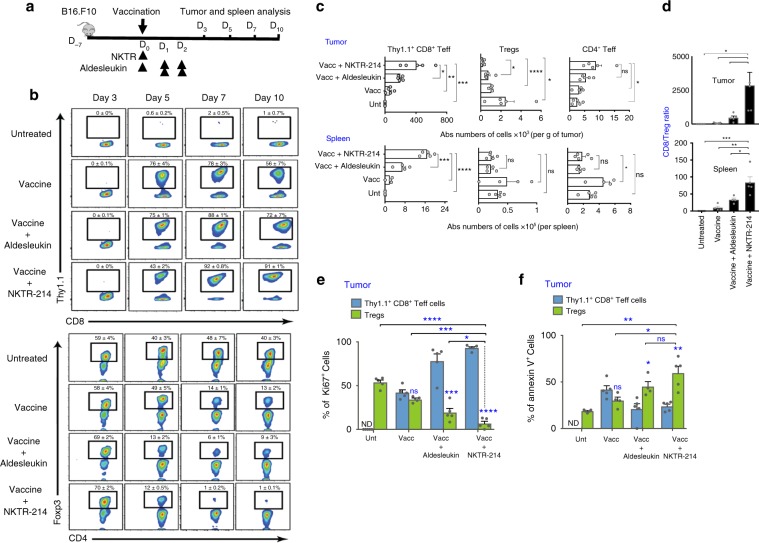
NKTR-214 selectively promotes survival and expansion of Teff and depletes Treg exclusively in tumor. **a**–**e** C57BL/6 mice bearing 7-day-old, s.c. B16.F10 tumors received pmel-1 T cells and vaccination followed by aldesleukin or NKTR-214. Splenic Thy1.1^+^ pmel-1 CD8^+^ Teff, CD4^+^ Teff, and Tregs were analyzed by flow cytometry on days 3, 5, 7, and 10 post treatment. **a** Experimental scheme. **b** Representative plots showing the level of CD8^+^ Teff (upper panel) and Tregs (lower panel) in tumor through time. **c** Absolute numbers of Thy1.1^+^ CD8^+^ Teff, Tregs, and CD4^+^ Teff in tumor (upper panel) and spleen (lower panel) analyzed on day 7 post treatment. Histograms showing **d** CD8/Treg ratio in tumor (upper panel) and spleen (lower panel). **e** Ki67 and **f** Annexin-V expression on tumor-infiltrating pmel-1 CD8^+^ Teff and Tregs on day 7 after treatment. Data represented as mean ± SEM (*n* = 4–5, *P < 0.05, **P < 0.01, ***P < 0.001, ****P < 0.0001, ns nonsignificant, unpaired *t* test).

While peripheral blood sampling offers a convenient window into immune responses after immunotherapy, tumor regression is mediated by intratumoral immune cells. We found that NKTR-214 enhanced both relative and absolute numbers of intratumoral gp100-specific CD8^+^ Teff, especially 7 and 10 days after gp100 peptide vaccination (Fig. 3a–c). Similar CD8^+^ Teff increases were observed in the spleen, indicating systemic Teff expansion (Fig. 3c). In contrast, we observed a progressive, drastic, and near-complete depletion of intratumoral Tregs but not of non-Treg CD4^+^ Teff (CD4^+^Foxp3^–^ T cells) in mice receiving vaccine and NKTR-214 (Fig. 3b, c). More modest Treg reductions were observed in mice receiving vaccine alone or vaccine with aldesleukin (Fig. 3b, c). Treg reduction was not observed in the spleens of the same mice, and the suppressive activity of splenic Tregs was comparable between the treatment groups (Fig. 3c and Supplementary Fig. 4a).

The CD8^+^ Teff expansion and reduction of intratumoral Tregs induced by vaccination and NKTR-214 resulted in high CD8/Treg ratios in tumors of both B16.F10 and CT26 models (Fig. 3d and Supplementary Fig. 3d). To understand how NKTR-214 simultaneously increased intratumoral CD8^+^ Teff and depleted intratumoral CD4^+^ Tregs, we analyzed T-cell proliferation and apoptosis. Overall, NKTR-214 strongly promoted proliferation and suppressed apoptosis of CD8^+^ Teff in tumor and spleen (Fig. 3e, f and Supplementary Fig. 4b, c). In contrast, NKTR-214 drastically reduced the proliferation and enhanced the apoptosis of CD4^+^ Tregs in tumor tissue, but not in spleen (Fig. 3e, f and Supplementary Fig. 4b, c). Thus, NKTR-214 shifted the balance between proliferation and apoptosis in the periphery versus in the tumor tissue, promoting expansion of intratumoral and peripheral CD8^+^ Teff while selectively depleting intratumoral but not peripheral Tregs. Both vaccine-activated CD8^+^ pmel-1 Teff and NKTR-214 were required to efficiently inhibit intratumoral Treg proliferation (Supplementary Fig. 4d).

### Intratumoral Tregs in mice and human post NKTR-214 treatment

**Fig. 4:**
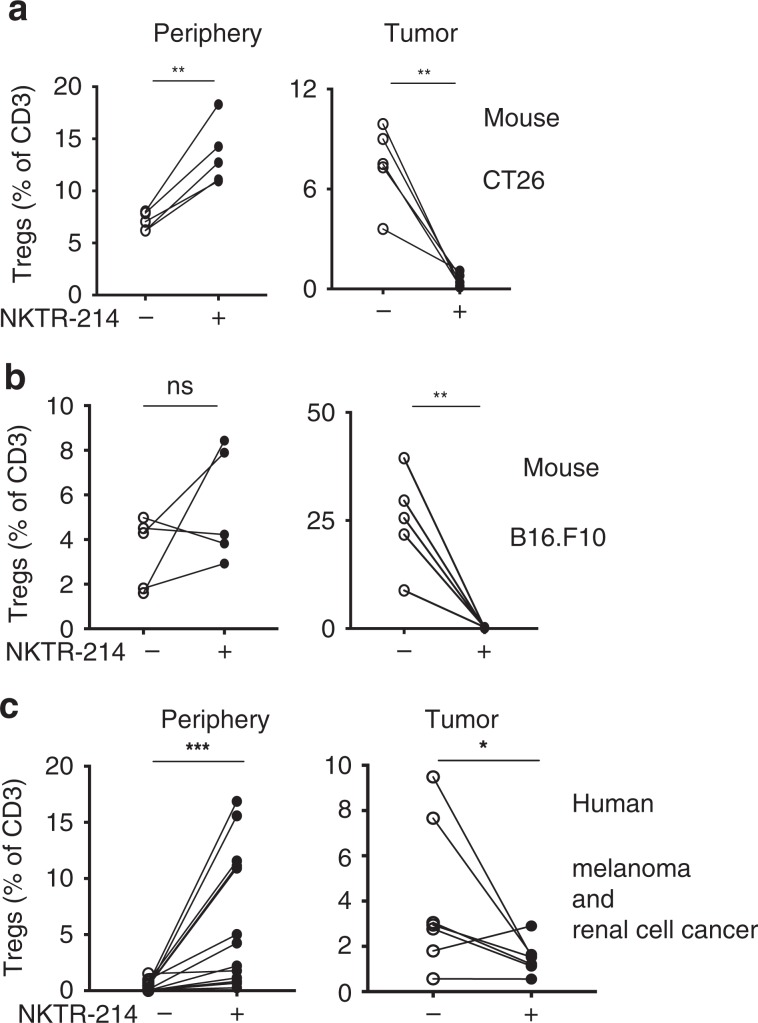
NKTR-214 induces intratumoral Treg depletion in mice and humans. **a** Mice bearing 4-day established s.c. CT26 tumors received either no treatment denoted by a minus sign (−) or NKTR-214 plus AH-1 vaccine, denoted by the plus sign (+); Treg levels in the spleen and tumor are shown on day 7 post treatment (*n* = 5, mean ± SEM, **P < 0.01, unpaired *t* test). **b** Mice bearing 7-day established s.c. B16.F10 tumors received pmel-1 T cells, and were either left untreated (−) or received NKTR-214 plus gp100 vaccine (+); Treg levels in the spleen and tumor on day 7 post treatment, are shown (*n* = 5, mean ± SEM, **P < 0.01, ns nonsignificant; unpaired *t* test). **c** Level of Tregs in blood (*n* = 13, mean ± SEM) and in tumor (*n* = 7, mean ± SEM) pre treatment (−) and 3 weeks post NKTR-214 treatment (+) in melanoma and renal cell carcinoma patients (*P < 0.05, ***P < 0.001; Wilcoxon-matched paired *t* test).

Animal models uniquely allow for experimental interventions that shed light on the basic mechanisms underlying cancer therapies; yet it is critical to confirm that these models represent real-life scenarios in patients. To understand whether intratumoral Treg depletion by NKTR-214 could also occur in patients treated with NKTR-214, we studied matched pre-treatment and post-treatment peripheral blood mononuclear cells (PBMC) and tumor biopsies from patients with melanoma and renal cell cancer (RCC) treated with NKTR-214. In mice, peripheral, splenic Treg levels were increased in most animals after NKTR-214 treatment in two different tumor models in two different mouse strains; similarly human Treg levels were increased in the circulation of most patients treated with NKTR-214 (Fig. 4a–c, left panels). In contrast, NKTR-214 therapy sharply reduced Treg levels in tumor tissue from the mice, as well as in tumors from patients (Fig. 4a–c, right panel). Within the inherent limitations of studying T-cell subset dynamics in tumor tissue from patients treated in a phase I clinical trial, these observations were not in contradiction with a mechanism of selective depletion of intratumoral Tregs by NKTR-214.

### CD8^+^ Teff-derived IFN-γ and TNF-α deplete intratumoral Tregs

**Fig. 5:**
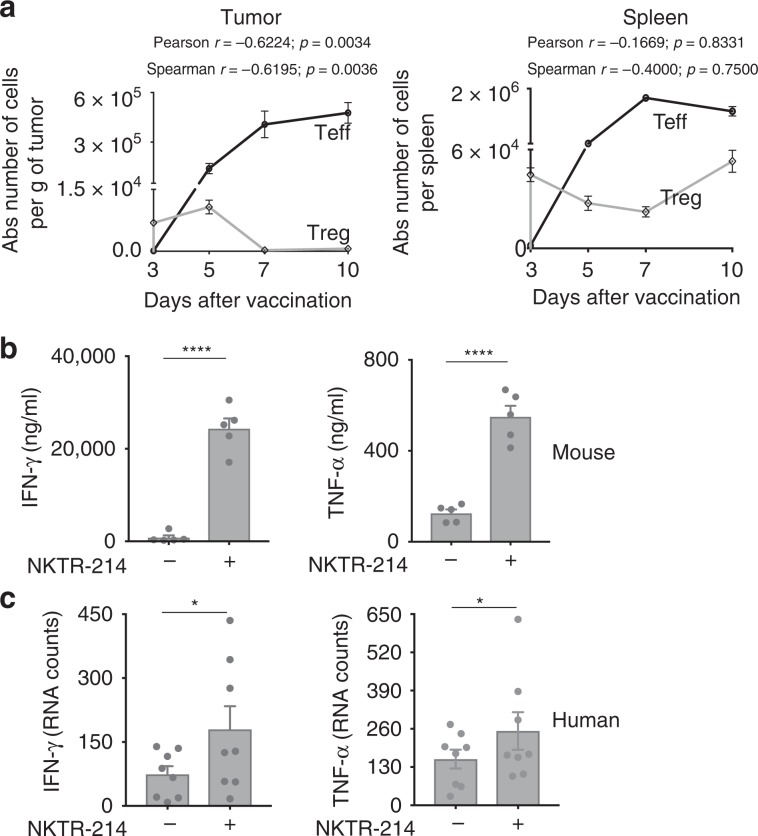
NKTR-214 induces intratumoral production of IFN-γ and TNF-α effector cytokines. **a** C57BL/6 mice bearing 7-day-old, s.c. B16.F10 tumors received pmel-1 T cells followed by treatment with NKTR-214 plus gp100 vaccination. Absolute numbers of pmel-1 Teff and Tregs in tumor and spleen on indicated days are plotted. Correlation between Teff and Treg numbers through time in each tissue was calculated, and Pearson’s and Spearman correlation coefficients are shown. **b** Level of IFN-γ and TNF-α protein in tumors of untreated or vaccine-NKTR-214-treated mice on day 7. Data represented as mean ± SEM (*n* = 5, ****P < 0.0001, unpaired *t* test). **c** Gene expression profiling of IFN-γ and TNF-α performed via NanoString in tumor biopsies from melanoma and renal cell carcinoma patients (*n* = 8), pre (−) and week 3 post NKTR-214 treatment (+). RNA counts are represented as mean ± SEM (*P < 0.05 ratio, paired t test).

**Fig. 6:**
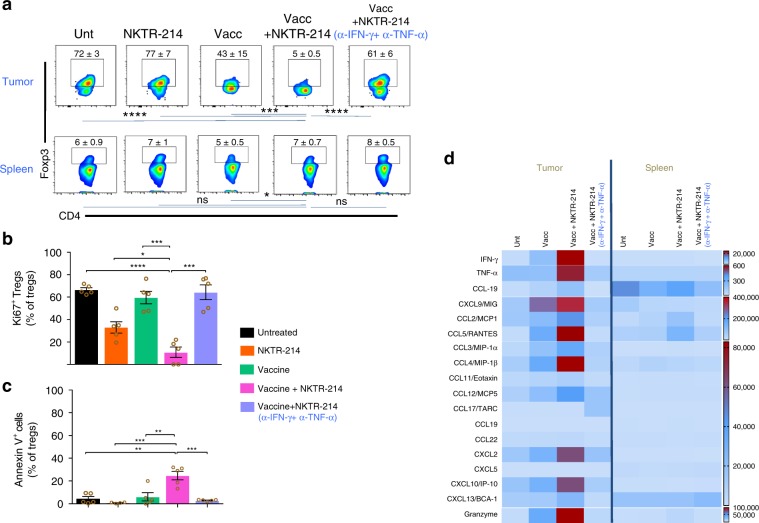
CD8^+^ Teff-derived IFN-γ and TNF-α synergize to deplete intratumoral Tregs. **a**–**d** C57BL/6 mice bearing 7-day-old, s.c. B16.F10 tumors received pmel-1 T cells, and were either left untreated or received gp100 peptide vaccine and/or NKTR-214. Where indicated, mice received neutralizing antibodies against IFN-γ and TNF-α on days 1, 2, 4, and 6. **a** Tregs in tumor (upper panel) and spleen (lower panel). **b** Expression of Ki67 and **c** Annexin V on intratumoral Tregs on day 7 post treatment. **d** Heat map depicting protein level of indicated cytokines and chemokines in tumor and spleen of mice 7 days after treatment. Color scale denotes levels of cytokines/chemokines in pg/ml. Data represented as mean ± SEM (*n* = 5, *P < 0.05, **P < 0.01, ***P < 0.001, ****P < 0.0001, ns nonsignificant, unpaired *t* test).

Since Treg depletion was observed in tumor tissue but not in the spleen of mice, and only occurred in the presence of vaccination-activated gp100-specific CD8^+^ Teff (Supplementary Fig. 4d), we hypothesized that intratumoral Treg depletion is mediated by CD8^+^ Teff responding to cognate tumor antigen highly expressed in the tumor tissue. Kinetic analysis demonstrated that the increase in intratumoral CD8^+^ Teff preceded intratumoral Treg depletion (Fig. 5a). Furthermore, in vivo depletion of CD8^+^ T cells in mice treated with vaccine and NKTR-214, completely abrogated intratumoral Treg depletion (Supplementary Fig. 4e), demonstrating a direct contribution of tumor-specific, CD8^+^ Teff to intratumoral Treg depletion. Since a prime function of CD8^+^ T cells is the production of effector cytokines, in particular IFN-γ and TNF-α, we sought to identify whether these cytokines might mediate intratumoral Treg depletion. We quantified the production of these cytokines in mouse (as protein) and human tumor tissue (as mRNA) pre and post NKTR-214 treatment. NKTR-214 administration increased the levels of both IFN-γ and TNF-α in B16.F10 tumor tissue and in human melanoma and RCC tumors (Fig. 5b, c). In contrast, spleens from the same mice contained only low levels of IFN-γ and TNF-α that were not enhanced after NKTR-214 treatment (Supplementary Fig. 5a). To directly examine the possible role of IFN-γ and TNF-α in intratumoral Treg depletion, we used monoclonal antibodies to neutralize both cytokines in mice that were treated with vaccine plus NKTR-214. Dual-cytokine neutralization completely restored intratumoral Tregs to levels observed in untreated mice (Fig. 6a), while single-cytokine neutralization was less effective (Supplementary Fig. 5b). However, no significant change was observed in the spleen (Fig. 6a), indicating that both the production of IFN-γ and TNF-α and consequent Treg depletion is restricted to tumor tissue, presumably due to local production of IFN-γ and TNF-α by CD8^+^ Teff encountering cognate gp100 tumor Ag in tumor but not in spleen. Vaccination and NKTR-214 treatment reduced both natural (neuropilin^hi^) and inducible (neuropilin^lo^) Tregs in tumor, with both Treg subsets restored upon cytokine neutralization (Supplementary Fig. 5c). Mechanistically, neutralization of IFN-γ and TNF-α fully restored the dampened proliferation of intratumoral Tregs and reduced their apoptosis in vivo (Fig. 6b, c). In contrast, cytokine neutralization significantly reduced numbers and proliferation of CD8^+^ Teff and CD4^+^ Teff, as well as numbers of NK cells in the tumor, indicating the specific depleting action of IFN-γ and TNF-α on Tregs versus Teff subsets (Supplementary Fig. 6a–e).

While reduced proliferation and enhanced apoptosis of Tregs can directly explain their reduced intratumoral numbers, IFN-γ and TNF-α can also impact the expression of many chemokines, including several that control Treg migration, and whose altered expression could also contribute to reduced intratumoral Treg numbers. In tumor tissue, as compared with control groups, vaccine plus NKTR-214 greatly enhanced the levels of IFN-γ and TNF-α, and multiple chemokines, including CCL2, CCL3, CCL4, CCL5, CXCL2, CXCL9, CXCL10, and CXCL13 (Fig. 6d and Supplementary Fig. 6f), while the expression of these chemokines was sharply reduced by neutralization of IFN-γ and TNF-α (Fig. 6d). These data indicate that IFN-γ and TNF-α deplete intratumoral Tregs despite strongly increasing local chemokine production. In addition, chemokines CCL17 and CCL22 that are known as key players in Treg migration remained unaltered by vaccination and NKTR-214 treatment. These findings suggest that intratumoral Treg depletion by IFN-γ and TNF-α is not due to altered chemokine expression, but instead directly caused by an altered balance between proliferation and apoptosis.

### IFN-γ and TNF-α directly inhibit Treg proliferation

**Fig. 7:**
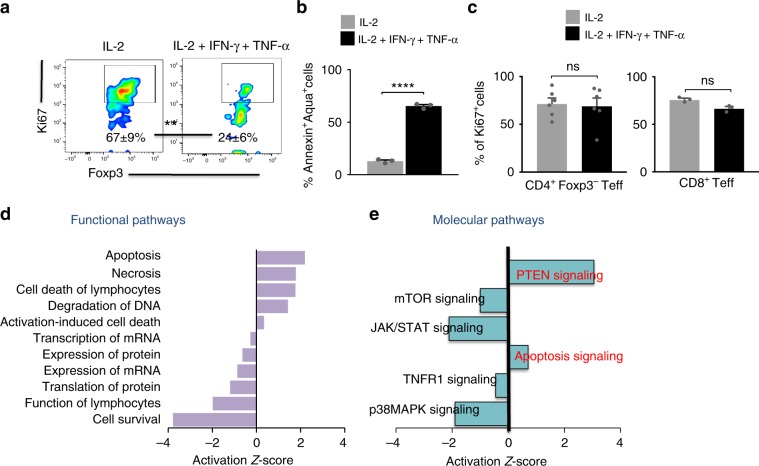
Impact of IFN-γ and TNF-α on specific signaling cascades in Tregs. **a**–**e** Splenic Tregs, non-Treg CD4^+^ T cells, and CD8^+^ Teff were cultured for 8 days in the presence of anti-CD3/anti-CD28 beads and IL-2 with or without IFN-γ and TNF-α. Culture supernatant was replaced biweekly with media containing fresh cytokines. **a** Expression of Ki67 and **b** expression of annexin V and viability dye aqua on Tregs. **c** Expression of Ki67 on CD4^+^ Foxp3^−^ Teff (left panel) and CD8^+^ Teff (right panel) is shown, analyzed on day 9. Data (mean ± SEM) is representative of 3–6 independent experiments (**p < 0.01, ****P < 0.0001; unpaired *t* test). **d**, **e** Reverse-phase protein array (RPPA) profiling of cultured Tregs on day 4. Enriched functional pathway and molecular pathway analysis on differentially expressed proteins (*P* value < 0.05 and fold change >1.25 or <1/1.25). Activation z score depicting up- or downregulated **d** functions and **e** signaling pathways in IL-2 and IFN-γ plus TNF-α-treated Tregs relative to Tregs treated with IL-2 alone. Data are from three experimental and three technical replicates.

To more rigorously determine whether IFN-γ and TNF-α act directly on Tregs or via other cells present in the tumor microenvironment, we studied the effect of IFN-γ and TNF-α on purified Tregs in vitro. Purified naive Tregs were activated with anti-CD3/CD28 beads and IL-2, and cultured with IFN-γ and/or TNF-α. As observed in vivo, IFN-γ and TNF-α dramatically reduced proliferation and increased apoptosis of purified Tregs in vitro (Fig. 7a, b). Mirroring the in vivo observations, both cytokines synergized to inhibit proliferation of purified Tregs (Supplementary Fig. 7a), but not purified CD8^+^ Teff or CD4^+^ Foxp3^–^ Teff (Fig. 7c). This inhibition of proliferation was not due to reduced expression of the IL-2R subunits CD25, CD122, and CD132 (Supplementary Fig. 7b). Indeed, IL-2-induced phosphorylation of the well-established IL-2R signaling molecule, STAT5, was not significantly altered by IFN-γ and TNF-α (Supplementary Fig. 7c). To gain a deeper understanding of the mechanism by which IFN-γ and TNF-α inhibit Treg proliferation, we examined molecular signaling pathways in protein lysates obtained from cultured Tregs by using reverse-phase protein array (RPPA). This analysis confirmed that in Tregs, IFN-γ and TNF-α inhibited molecular pathways related to cellular survival, while activating pathways related to apoptosis and necrosis (Fig. 7d, e). Specifically, we found increased expression of known IFN-γ-induced pro-apoptotic molecules such as Caspase-3 and STAT1^24,25^ (Supplementary Fig. 7e, f). Simultaneously, we observed decreased expression of the anti-apoptotic molecules Bcl-XL and C-myc, known to be downregulated by IFN-γ and TNF-α in other cell types^24–28^ (Supplementary Fig. 7e, f, Supplementary Table 1). IFN-γ and TNF-α also suppressed signaling in major pathways of activation and proliferation, such as pAkt-mTOR, JAK/STAT, and MAPK, while PTEN, a known inhibitor of the pAkt-mTOR axis, was upregulated (Fig. 7e and Supplementary Fig. 7d). Together, these results suggest that IFN-γ and TNF-α synergize to regulate specific pathways of T-cell survival and apoptosis, resulting in reduced Treg proliferation.

## Discussion

T cells mediate the response to PD-1 and CTLA-4 CPI therapy, as well as adoptive chimeric antigen receptor (CAR) T-cell therapy and cancer vaccines, positioning T-cell growth factors such as IL-2 as ideal combination partners. However, HD IL-2 therapy is accompanied by substantial toxicity, and can promote the expansion of CD4^+^Foxp3^+^ Tregs that impede antitumor immunity. Indeed, a 4–5-fold increase in Tregs was documented in melanoma patients post HD IL-2 therapy, and increased expansion of ICOS^+^ Tregs correlated with disease progression in these patients^10^. To overcome these negative features of IL-2, NKTR-214 is an IL-2 prodrug specifically engineered for a long in vivo half-life and preferential binding to IL-2Rβγ over IL-2Rα^15,14^. Our preclinical data demonstrate potentiation of PD-1 CPI therapy by NKTR-214, resulting in a higher response rate, prolonged disease control, and more complete responses in a variety of histologies, including melanoma, bladder cancer, lung cancer, and breast cancer. Similarly, we observed potent synergy of NKTR-214 with anticancer vaccination. Consequently, multiple clinical trials of NKTR-214 in combination with anti-PD-1, anti-CTLA-4, or other agents in multiple cancer histologies are currently underway (e.g., NCT02983045, NCT03138889, NCT03435640, NCT03635983).

While NKTR-214 induced bursts of proliferation in both Teff and Treg, systemic levels of Teff remained elevated over multiple courses of treatment, while systemic Tregs returned to baseline between treatment cycles. While intratumoral Teff increased, intratumoral Treg levels dropped precipitously to nearly undetectable. Systemic Treg depletion could be detrimental to a patient, potentially placing them at risk for a variety of autoimmune phenomena, particularly while on concomitant immunostimulatory CTLA-4 and/or PD-1 CPI therapy. Therefore, a strategy for preferential depletion of intratumoral Tregs may be extremely useful, given their well-appreciated suppressive effect on antitumor immunity in mouse and man^29,30^. While in mice, anti-CTLA-4 mAbs can potently deplete intratumoral Tregs through direct binding to CTLA-4^+^ Tregs and their subsequent depletion by macrophage-mediated effector functions^31^, this mechanism does not appear to be operative in patients treated with the FDA-approved human anti-CTLA-4 mAb, ipilimumab^32^. In contrast, NKTR-214-induced intratumoral Treg depletion was observed across different tumor models in two different strains of mice, with similar trends also observed in tumors from patients with melanoma and renal cell cancer treated with NKTR-214.

The intratumoral depletion of Tregs was accompanied by their decreased proliferation and increased apoptosis. Mechanistically, these effects required both activated CD8^+^ Teff and NKTR-214 in vivo. Diminished intratumoral Treg numbers were completely restored upon in vivo CD8^+^ T-cell depletion and also by in vivo neutralization of IFN-γ and TNF-α. Together, these results indicate that vaccination-induced CD8^+^ Teff cells that were sustained and further expanded by NKTR-214, released IFN-γ and TNF-α to drive efficient intratumoral Treg depletion. Treg depletion was observed only in the tumor, where Teff can recognize cognate gp100 tumor antigen, and produced IFN-γ and TNF-α. In contrast, Tregs were not depleted in the spleen, where despite large numbers of Teff, little IFN-γ and TNF-α were detected, presumably due to the local absence of cognate gp100 tumor antigen. Together, these findings suggest a pivotal role of CD8+ Teff as a source of IFN-γ and TNF-α; however, in the complex intratumoral immune microenvironment, a contribution of other immune cells such as CD4+ T cells and NK cells in mediating NKTR-214-associated Treg depletion and antitumor effect cannot be excluded.

The correlation of increased ratios of intratumoral CD8^+^ Teff to CD4^+^ Tregs with tumor control after immunotherapy is well appreciated^31^. However, increases in this ratio are typically attributed to increases in CD8^+^ T cells rather than to decreases in CD4^+^ Tregs. Nevertheless, a few reports have demonstrated decreases in intratumoral Tregs during immunotherapy; for example, Liakou et al. reported that bladder cancer tissue from patients treated with anti-CTLA-4 mAb (ipilimumab) became infiltrated with IFN-γ-producing Teff cells while CD4^+^Foxp3^+^ Treg numbers dwindled; however, the mechanism underlying this Treg reduction was not elucidated^33^. Similarly, two animal studies showed that IFN-γ could either render Tregs functionally fragile without affecting their numbers in vivo^34^, or reduce Treg proliferation in vitro^35^, although the change was modest and similar to what we observed with IFN-γ alone; a synergistic role of TNF-α was not described. Possible synergistic activity of these two effector cytokines, commonly produced together by Teff in response to cognate Ag, on Treg proliferation and survival has, to our knowledge, not been previously explored. Since the tissue microenvironment and accompanying factors that affect Tregs can vary widely between tumor types in mice and men, it will be important to understand the precise conditions that modulate the effects of IFN-γ and TNF-α on intratumoral Tregs. While this report focuses specifically on the mechanism of action of NKTR-214, it is conceivable that other potent immunotherapies that induce IFN-γ and TNF-α could have similar depleting effects on intratumoral Tregs. Future studies are also needed to examine whether Treg depletion by effector cytokines is a generalized phenomenon that temporarily and locally reduces untoward immunosuppression by Tregs in pathogen-infected tissues, and whether it plays a role in exacerbating or maintaining autoimmune disease.

Antiproliferative and pro-apoptotic synergy of IFN-γ and TNF-α have been reported in various non-Treg cell types, including colon carcinoma, neuroblastoma, salivary ductal cell lines, and pancreatic beta cells, and the specific signaling cascades are partially defined^27,36–38^. Our proteomic analysis of cultured Tregs confirmed upregulation of growth-inhibitory and apoptotic molecules STAT1 and Caspase-3 downstream of the IFN-γ receptor (IFN-γR), and downregulation of proliferation-associated molecules C-myc and NF-κB downstream of the TNF-α receptor (TNFR)^24–26^. Further, major signaling pathways MAPK, JAK/STAT, and PI3K–AKT–mTOR downstream of IL-2R and TCR engagement^39^, were downmodulated in Tregs by IFN-γ and TNF-α treatment. Consistent with this, we found that IFN-γ and TNF-α increased expression of the PI3K-negative regulator, PTEN, as previously reported for macrophages and human leukemic cells^40,41^. PI3K inhibitors have also shown promising antitumor effects associated with selective inhibition of activation and proliferation of Tregs, while having no or little effect on CD4^+^CD25^–^ and CD8^+^ T cells^42–44^. These findings bear an interesting resemblance to our observations on Treg-specific depletion by IFN-γ and TNF-α. Future studies will identify the contribution of individual signaling pathways to inhibition of Treg proliferation, and it will be interesting to study whether effector cytokines such as IFN-γ and TNF-α can function as natural inhibitors of the PI3K–AKT–mTOR pathway selectively in Tregs.

In summary, we report therapeutic synergy of NKTR-214 with PD-1 and CTLA-4 CPI therapy and with vaccination in multiple tumor models and mouse strains, accompanied by dramatically increased systemic and intratumoral CD8^+^ T-cell responses. The observation that NKTR-214 treatment mediated selective Treg depletion of intratumoral but not of peripheral Tregs suggests that in patients with cancer, NKTR-214-containing regimens could increase tumor control without exacerbating systemic inflammation. Thereby, NKTR-214-based treatment could prevent systemic immune alterations, which might occur with therapeutic strategies that result in systemic Treg depletion. Clinical trials are underway for patients with a variety of cancers who receive NKTR-214/bempegaldesleukin in combination with anti-PD-1 (nivolumab and pembrolizumab), anti-CTLA-4 (ipilimumab), and other agents.

## Methods

### Mice and cell lines

Animals were housed under specific pathogen-free conditions as per the national animal testing regulations. All the murine experiments performed in this study were approved by the Institutional Animal Care and Use Committee (IACUC) of the University of Texas MD Anderson Cancer Center. Six-to-eight-week-old female C57BL/6 or BALB/c mice were purchased from Charles River Laboratory. Pmel-1 TCR transgenic mice on a C57BL/6 background (The Jackson Laboratory, Bar Harbor, ME) were crossed with CD90.1 congenic mice to yield pmel-1^+/+^ × CD90.1^+/+^ mice (hereafter referred to as pmel-1 mice)^19^. All murine cell lines were obtained from ATCC and were maintained in complete medium, including RPMI 1640 with 10% FBS, 100 µg/ml streptomycin, and 100 µg/ml penicillin (Life Technologies, Carlsbad, CA).

Eight tumor models were evaluated in four different mouse strains: C57BL/6 strain for LLC (lung), Pan02 (pancreatic), and B16.F10 (melanoma); C3H strain for MBT-2 (bladder); BALB/c strain for CT26 (colon), H22 (liver), and EMT6 (breast); FVB strain for BR5FVB (ovarian). The latter cell line contains a BRCA and akt mutation similar to the genetics of many human serous ovarian tumors^45^.

### Tumor treatments

Eight different tumor cell lines (LLC, Pan02, B16F10, MBT-2, CT26, H22, EMT6, and BR5FVB) were inoculated subcutaneously in syngeneic mice, and tumors were grown to 100 mm^3^ prior to treatment. Animals were randomized to each of four groups to receive vehicle, single agent NKTR-214 (0.8 mg per kg q9dx3, NEKTAR Pharmaceuticals, CA), single agent anti-PD-1 (RMP1–14 at 200 μg per mouse, 2× per week, BioXcell, NH), or the combination at the same dose regimen. In all tumor models except CT26, NKTR-214 treatment was initiated 4 days after the start of anti-PD-1 therapy. CT26 tumors were treated on day 7 after tumor induction with either anti-CTLA-4 (4F10 at 100 μg per mouse, 2× per week, BioXcell, NH) or anti-PD-1 (RMP1–14 at 200 μg per mouse, 2× per week), or together with or without 0.8 mg per kg NKTR-214 every 8 days. *n* = 7–10 animals per group. To compare antitumor efficacy of aldesleukin with NKTR-214, 6-day-old CT26 tumor-bearing mice were treated with vehicle or anti-PD-1 (RMP1–14 at 200 μg per mouse, q4dx5, BioXcell, NH) alone; aldesleukin (0.5 mg per kg, qdx5 two cycles) or NKTR-214 (0.8 mg per kg, q9dx3, NEKTAR Therapeutics, CA) alone or with anti-PD-1.

B16.F10 tumor-bearing C57BL/6 mice were vaccinated 7 days after tumor implantation, and treated with or without IL-2 or with or without NKTR-214 as indicated. Each mouse was vaccinated s.c. on each flank with 50 µg of heteroclitic mouse gp100_25–33_ (hgp100) peptide KVPRNQDWL (Peptides International, Louisville, KY) formulated in l-Tyrosine microparticles^46^ along with 50 µg s.c. of anti-CD40 antibody (clone FGK4.5/FGK45, BioXcell, New Hampshire) and 50 mg per mouse Imiquimod cream 5% (Fougera, Melville, NY). In order to track tumor-specific T-cell responses, each mouse received 80,000 naive splenic Thy1.1 congenic pmel-1 CD8 T cells i.v. on the day of vaccination. Mice in indicated treatment groups received aldesleukin (human rIL-2; Prometheus laboratories Inc., CA) at 100,000 IU once on the day of vaccination and twice a day, on the 2 following days, or as indicated. NKTR-214 was given once, i.p. in different doses (0.2 mg per kg, 0.1 mg per kg, and 0.05 mg per kg). Unless specified otherwise, these regimens of aldesleukin and NKTR-214 were repeated every 8 days. Tumor size was measured with calipers and expressed as the product of perpendicular diameters. Mice were sacrificed when tumor size reached ≥200 mm^2^. Where indicated, mice received i.p. injection of 100 µg of each of anti-IFN-γ Clone, XMG1.2 or anti-TNF-α Clone, XT3.11 or anti-CD8 Clone, YTS 169.4 (BioXcell, NH) on days 1, 2, 4, and 6 post vaccination.

### T-cell receptor clonality and tumor density

The murine CT26 tumor model was used for assessing T-cell density and clonality. T-cell density was measured in the tumor microenvironment by DNA content. The TCR Vβ and Jβ usage was determined utilizing the ImmunoSEQ platform from Adaptive Biotechnologies after treatment with nothing, 0.8 mg per kg NKTR-214, anti-PD-1 mAb (RMP1–14 200 μg twice weekly), or their combination (*n* = 4 mice/group).

### In situ intratumoral T-cell analysis

Mice bearing established 100 mm^3^ B16F10 tumors were administered vehicle, NKTR-214 (0.8 mg per kg, q9dx3), fingolimod (5 μg per mouse, qd), or their combination, *n* = 10/group. Fingolimod (FTY720, Sigma, St. Louis, MI, USA) was administered for 7 days prior to the start of NKTR-214 treatment to ensure reduced lymphocyte counts in blood. CD8^+^ T cells were quantified in tumor and blood by flow cytometry.

### Histology and immunofluorescence staining

Tumors were collected 30 and 60 days after initiation of treatment, embedded in O.C.T, frozen, sectioned, and stained with hematoxylin and eosin (H&E). Methanol-fixed sections were stained with rabbit monoclonal anti-gp100 antibody (Abcam, MA) followed by goat anti-rabbit Alexa Fluor 488 (Invitrogen, Thermo Fisher, CA) antibody and DAPI nuclear stain before photography with a Leica digital camera DFC 3000 G and Leica DMi8 immunofluorescence microscope, and processed by Leica Application Suite X software.

### Cytokine/chemokine assay

On days 3, 5, 7, and 10 post vaccination, tumor samples were weighed, and tumor and spleen samples were mechanically disrupted in 1 ml of ice-cold PBS per sample. Samples were then centrifuged for supernatant collection. The cytokines/chemokines in the supernatant were measured using Milliplex mouse cytokine/chemokine panel (R&D systems, MN) according to the manufacturer’s instructions. Fluorescence signal was measured on a Luminex 100/200 system, and data were analyzed using Excel software.

### Tissue preparation and flow cytometry

Single-cell suspensions from spleen and tumor were prepared in PBS with 10% FCS and 2 mM EDTA (Sigma-Aldrich, Missouri) by mashing tissue against the surface of a 40-μm cell strainer using a plunger of 3-ml syringe (BD Biosciences, CA). Lymphocytes from tumor tissue were enriched on a Ficoll gradient (Histopaque 1119, Sigma-Aldrich, Missouri), while RBCs (red blood cells) were removed from spleen samples using a hypotonic lysis buffer (StemCell Technologies, MA). Blood from mice was drawn by tail-snipping method, and RBCs were lysed using ACK lysis buffer (Thermo Fisher Scientific, MA), to get peripheral blood mononuclear cell (PBMC) suspension. Flow cytometric stains were performed with Foxp3 and Annexin-V staining kits (eBiosciences, Affymetrix, CA), LIVE/DEAD ^TM^ fixable aqua dead cell stain kit (Thermo Fisher Scientific, CA), and antibodies against CD25 (clone PC61), Ki67 (clone B56), CD3e (17A2), and STAT5 (PY694) from BD Biosciences; CD8 (clone 53-6.7), CD4 (clone GK1.5), CD122 (Clone TM-β1), and CD132 (clone TVGm2) from Biolegend, CA; CD90.1 (clone HIS51) and Foxp3 (clone FJK-16s) from eBiosciences (Thermo Fisher, MA); CD304 (Neuropilin; clone 3DS304M) from Invitrogen, CA. For intracellular staining, cells were fixed and stained for Ki67 and Foxp3 using Foxp3 staining kit from eBiosciences (Affymetrix, CA). Phosphorylated (p)STAT5 staining was done using transcription phosphor buffer set (BD Biosciences, CA) following the manufacturer’s protocol. All staining antibodies were used at 1:50 or 1:100 or 1:200 dilution depending on the experiment. Sample acquisition was performed on a BD Fortessa flow cytometer using FACS Diva software followed by data analysis with FlowJo software.

### In vitro cell culture

Tregs (CD4^+^ CD25^hi^), CD4^+^ Foxp3^–^ Teff, and CD8^+^ Teff cells were obtained from spleens of naive C57BL/6 mice and cultured in complete medium (RPMI 1640 supplemented with 10% heat-inactivated FBS, 100 μg per ml penicillin, and 100 μg per ml streptomycin) in a 96-well U-bottom plate in the presence of anti-CD3/anti-CD28 beads (Thermo Fisher, CA), and 1000 IU per ml rhIL-2 with or without 50 ng per ml rmIFN-γ and 50 ng per ml rmTNF-α (both from R&D systems, MN). For analysis of Ki67, CD25, CD122, CD132, and Annexin-V cells were cultured for 8 days, and the supernatant was replaced biweekly with media containing fresh cytokines. For reverse-phase protein array (RPPA) analysis, Tregs were cultured for 3 days, and protein lysate was prepared on day 4. For PTEN analysis by RPPA, cultured Tregs were washed and rested in complete medium overnight upon 3 days of culture, followed by addition of 50 ng per ml rmIFN-γ and 50 ng per ml rmTNF-α for 6 h, and 1000 IU per ml of rhIL-2 for the last 30 min of culture followed by protein lysate preparation.

C57BL/6 mice bearing 7-day established s.c. B16.F10 tumors received pmel-1 T cells and gp100 peptide vaccine with or without NKTR-214. CD4^+^ CD25^–^ T cells and CD4^+^ CD25^hi^ Tregs were sorted on day 7 from splenocytes of either untreated mice or mice that received vaccine or vaccine plus NKTR-214. CD4^+^ CD25^–^ responder T cells were CFSE (Thermo Fisher, MA) labeled by incubating cell suspension in 5 μM of CFSE for 20 minu at room temperature. Cells were then washed twice with media containing FBS. Labeled cells were rested for 15 min and then cultured for 72 h in a 1:1 ratio with either unlabeled responder CD4^+^ T cells or Tregs from the same treatment group, in the presence of anti-CD3/anti-CD28 beads and IL-2. Percentages of non-proliferated CSFE-labeled Teff were analyzed by flow cytometry using Fortessa X-20 (BD Biosciences, CA).

To analyze the effect of IFN-γ and TNF-α treatment on phosphorylated (p)STAT5 in Tregs, CD4^+^ CD25^hi^ Tregs were freshly sorted from the spleen of WT mice and cultured in a 96-well U-bottom plate for 6 h in the presence of anti-CD3/CD28 microbeads (Thermo Fisher, CA), 50 ng per ml of rmIFN-γ and 50 ng per ml of rmTNF-α, followed by addition of 1000 IU per ml rhIL-2 during the last 30 min of culture. Cells were then subjected to pSTAT5 analysis by flow cytometry.

### Reverse-phase protein array analysis

Reverse-phase protein array (RPPA) assays were carried out using defined methods with minor modifications^47–49^. Protein lysates were prepared from Tregs cultured for 3 days in the presence of anti-CD3/CD28 with 1000 IU per ml rhIL-2 with or without 50 ng per ml rmIFN-γ and 50 ng per ml rmTNF-α. Lysates were spotted onto nitrocellulose-coated slides (Grace Bio-labs) using an array format of 960 lysates/slide (2880 spots/slide), processed as described, and probed with a set of 216 antibodies against total phosphoproteins (supplementary information), using an automated slide stainer Autolink 48 (Dako). Each slide was incubated with one specific primary antibody, and a negative control slide was incubated with antibody diluent instead of primary antibody. Primary antibody binding was detected using a biotinylated secondary antibody followed by streptavidin-conjugated IRDye680 fluorophore (LI-COR Biosciences). Total protein content of each spotted lysate was assessed by fluorescent staining with Sypro Ruby Protein Blot Stain according to the manufacturer’s instructions (Molecular Probes).

Fluorescence-labeled slides were scanned on a GenePix 4400 AL scanner, and images were analyzed with GenePix Pro 7.0 (Molecular Devices). Total fluorescence signal intensities of each spot were obtained after subtraction of the local background signal for each slide, and were then normalized as described^47^. The median of the triplicate experimental values (normalized signal intensity) is taken for each sample for subsequent statistical analysis. We determined significantly changed proteins between experimental groups by employing Student’s *t* test (significant for *p* < 0.05), and requesting a fold change of at least 1.25×^47^. The data were further analyzed using Microsoft Excel, Inguinity pathway analysis (Qiagen Bioinformatics), and Cytoscape.

### Patient sample analysis

Melanoma or renal cell carcinoma (RCC) patients were treated with 0.003 or 0.006 or 0.009 mg per kg body weight of NKTR-214 every 3 weeks (q21d), and blood and tumor biopsies were obtained on the first day of treatment (−) and after 3 weeks post NKTR-214 treatment (+). This study is detailed under identifier NCT02869295 on www.clinicaltrial.gov. The study protocol was approved by Institutional Review Board (IRB) of MD Anderson Cancer Center. All patients provided written informed consent before any protocol-specified procedures. Primary outcomes of the study were to evaluate safety, maximum tolerated dose (MTD), recommended phase 2 dose (RP2D), and antitumor clinical activity. Secondary outcomes were to characterize pharmacokinetics and pharmacodynamics, and blood and tumor analysis to detect immunological changes. Whole blood was collected and processed to obtain PBMCs, while tumor biopsies were disaggregated using the BD Medimachine system according to the manufacturer’s instructions. Suspension of cells from either blood or tumor were stained with fluorochrome-conjugated, anti-human antibodies CD3 FITC (SK7), CD4 ACP-H7 (RPA-T4), and CD8 PB (RPA-T8); all antibodies were from BD and Foxp3 eFluor 450 (PCH101), from eBiosciences, San Diego, CA. Intracellular Foxp3 and cell-surface CD25^hi^ were used to discriminate between conventional CD4^+^ T cells and CD4^+^ Treg cells. Live/DEAD Fixable Aqua Dead Cell Stain Kit (Invitrogen, Carlsbad, CA) was used to discard dead cells. Briefly, cells were incubated with antibodies for 30 min, then washed, and fixed with paraformaldehyde 1% (Sigma). Intracellular staining for Foxp3 was performed using Foxp3 Fixation Kit (eBioscience, Thermo Fisher, MA). Stained cells were acquired on LSRII Fortessa and Canto II (BD Biosciences, CA). FlowJo software (Tree Star, Ashland, OR) was used to analyze data. Blood analysis was done in *n* = 4 melanoma and *n* = 9 RCC patients, while tumor analysis was performed in *n* = 3 melanoma and *n* = 4 RCC patients.

For analysis of IFN-γ and TNF-α mRNA in tumor tissue, NanoString nCounter gene expression assay was performed on RNA extracted from tumor biopsies obtained from melanoma (*n* = 4) and RCC patients (*n* = 4) using the RNeasy Micro Kit (Qiagen, CA) followed by hybridization with code sets, and then scanning using the nCounter Digital Analyzer as per the manufacturer’s instructions (NanoString Technologies, Seattle, WA). Gene expression was analyzed using Human PanCancer Immune Profiling Panel followed by analysis using nSolver software 4.0 (NanoString Technologies). The expression levels of each gene were normalized to those of control genes.

### Statistical analysis

All statistical analyses were performed using GraphPad Prism version 6 for Windows (GraphPad Software, San Diego, CA). All results are expressed as the means ± SEM. Mouse sample group sizes were *n* = 5, unless otherwise indicated. Data were analyzed using paired or unpaired two-tailed *t* tests or one-way analysis of variance (ANOVA), and differences were considered significant at *P* < 0.05. Figures denote statistical significance of *P* < 0.05 as *, *P* < 0.01 as **, *p* < 0.001 as ***, and *p* < 0.0001 as ****. Survival experiments utilized log-rank Mantel–Cox test for survival analysis. All animal experiments were repeated at least once, and in vitro experiments at least thrice with comparable results.

### Reporting summary

Further information on research design is available in the Nature Research Reporting Summary linked to this article.

## Supplementary information

Supplementary Information

Reporting Summary

## Data Availability

The raw data underlying the main Figs. 1b, c, 2c, d, 3b–f, 6a–d, and 7a–c as well as Supplementary Figs. 1a, d, 2b, c, f, 3d, 4b–e, 5a–c, 6a–f, 7a–d of this study are provided as source data file. The associated data for Fig. 7d, e, Supplementary Fig. 7e, f are provided in Supplementary Table 1. Additional data are available from the corresponding author upon reasonable request.

## Source data

Source Data
