# Supplementary material for: Bempegaldesleukin selectively depletes intratumoral Tregs and potentiates T cell-mediated cancer therapy
Source: Nat Commun. 2020 Jan 31;11:661. doi: 10.1038/s41467-020-14471-1 (PMC6994577; doi:10.1038/s41467-020-14471-1)
Supplement: Supplementary file 1 — Supplementary Information [file 41467_2020_14471_MOESM1_ESM.pdf]

Supplementary information

Bempegaldesleukin selectively depletes intratumoral Tregs and potentiates T cell-mediated cancer therapy

Sharma et al.

Supplementary Fig. 1

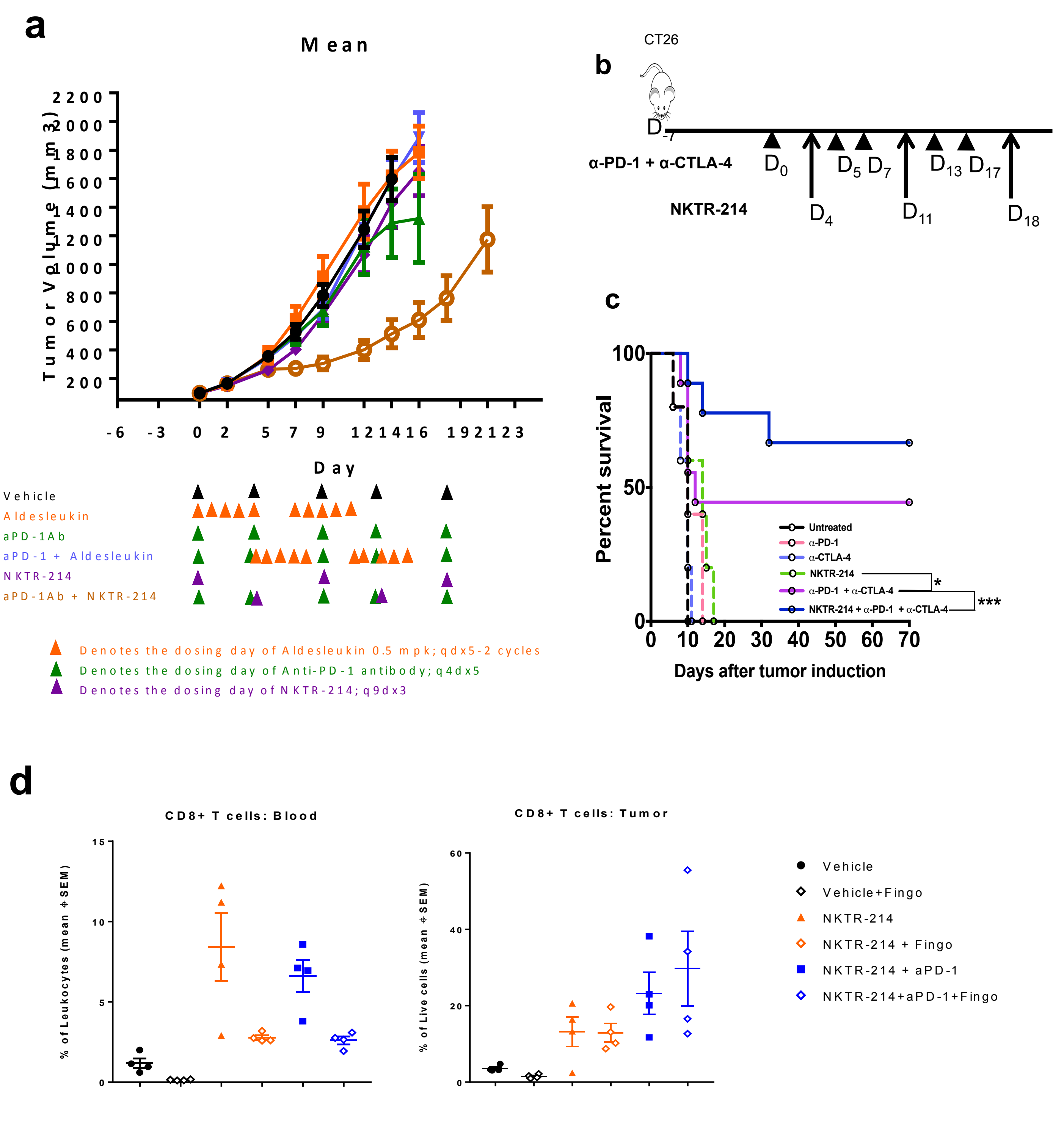

Supplementary fig 1. (**a**) Mice bearing 6 days old established CT26 tumors treated with vehicle or anti-PD-1 (q4dx5) alone, NKTR-214 (q9dx3) or aldesleukin (qdx5 two cycles) alone or in combination with anti-PD-1. Mean tumor volume (mm^3) in different treatment groups is shown. **(b-c)** BALB/C mice bearing 7 d, palpable s.c. CT26 tumors left untreated or received anti-PD-1 and anti-CTLA-4 combination therapy i.p. on day 0, 5,7,13,17 with or without NKTR-214 (i.p.) on day 4 with repeat dosing every 7 days. **(b)** Experimental scheme. **(c)** Kaplan-Meier survival curves are shown, each with 5 to 10 mice per group. *P < 0.05, ***P < 0.001, log-rank test. (**d**) Mice bearing established 100 mm^3 CT26 tumors were administered either vehicle, NKTR-214 (0.8mg/kg, q9dx3), fingolimod (5ug/mouse, qd) or the combination; n=10/group. Fingolimod was started 7 days prior to start of treatment to ensure reduced lymphocyte counts in blood. $CD8^+$ T cells in blood and tumor tissue were quantified by flow cytometry 10 days later.

Supplementary Fig. 2

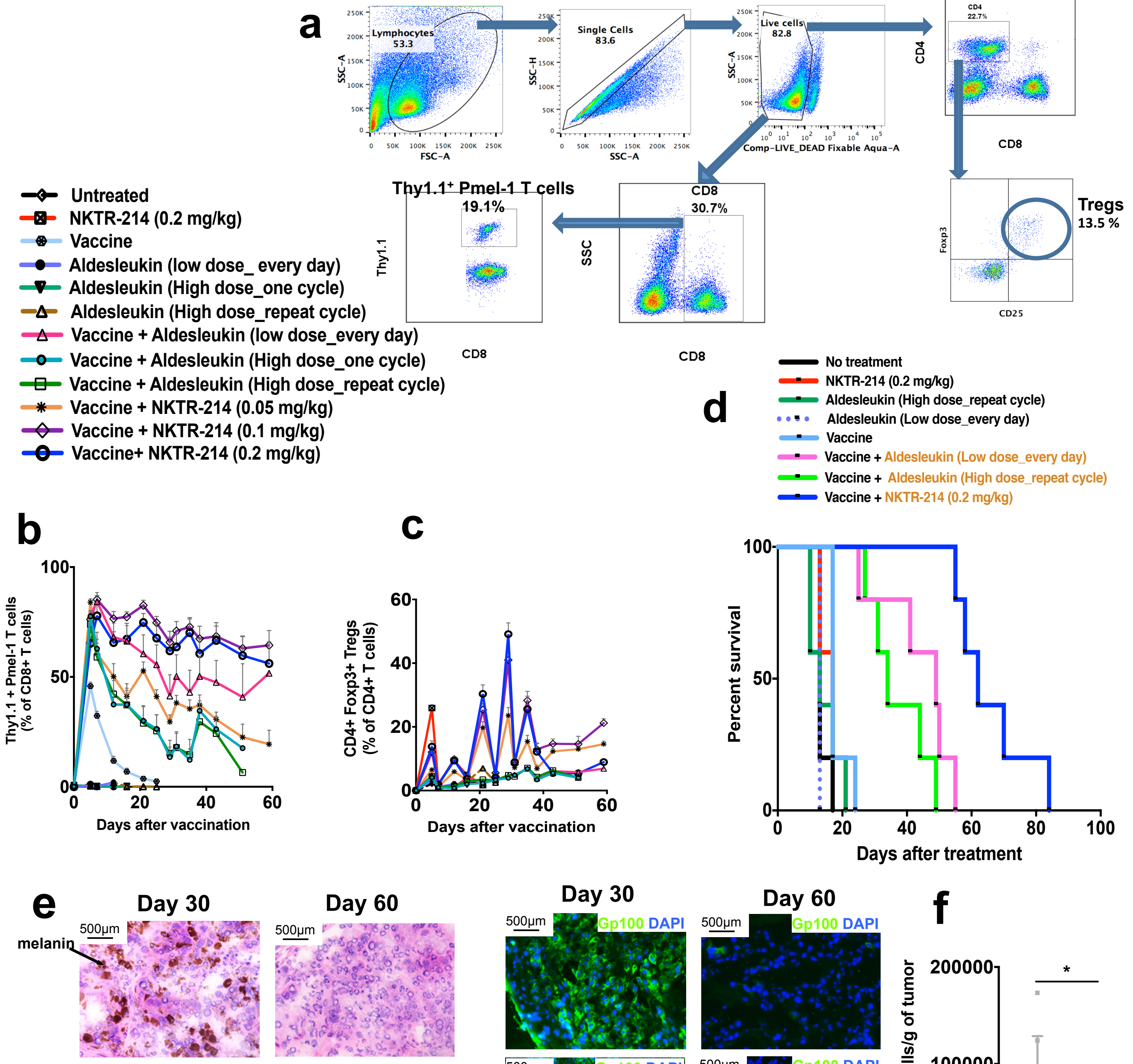

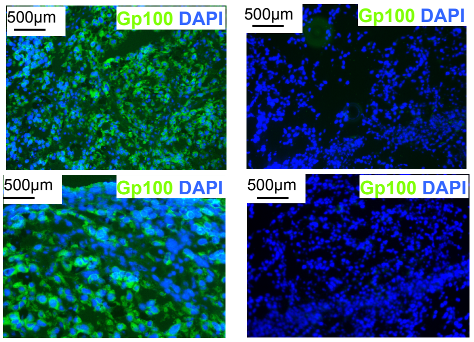

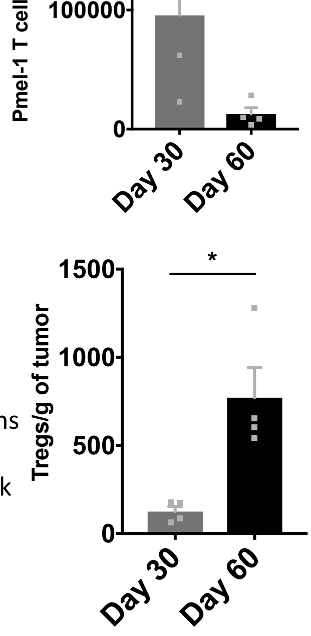

Supplementary fig 2. (a-f) C57BL/6 mice bearing 7-day established, s.c. B16.F10 tumors received pmel-1 T cells followed by gp100 vaccine and/or NKTR-214 as indicated or aldesleukin at different dosing regimen; low dose_every day (62,500 IU/mouse), high dose_one cycle (100,000IU on Day1, 200,000IU on day 2 and day 3) or high dose_repeat cycle (100,000IU on Day1, 200,000IU on day 2 and day 3, followed by repetition of dosing cycle every day 8 days).

(**a**) Gating strategy for flow cytometry analysis, (**b**) Level of Thy1.1⁺ CD8⁺ Teff and (**c**) proportions of Tregs were analyzed in blood of indicated groups at different time points, are shown (n=5, mean± SEM). (**d**) Kaplan-Meier survival curves, each with 5 mice per group. **P < 0.01, log-rank test. (**e-f**) B16.F10 tumors were isolated on day 30 and 60 from mice treated as in (a) with vaccine and NKTR-214 (0.2 mg/kg every 8 days) and (**e**) stained for gp100 with H&E counterstain. **(f)** Histograms depicting numbers of Tregs (right panel) and Thy1.1⁺ CD8⁺ pmel-1 Teff (left panel) in day 30 and day 60 tumors. Data is represented as mean± SEM (n = 4, *P<0.05, unpaired t-test.).

Supplementary Fig. 3

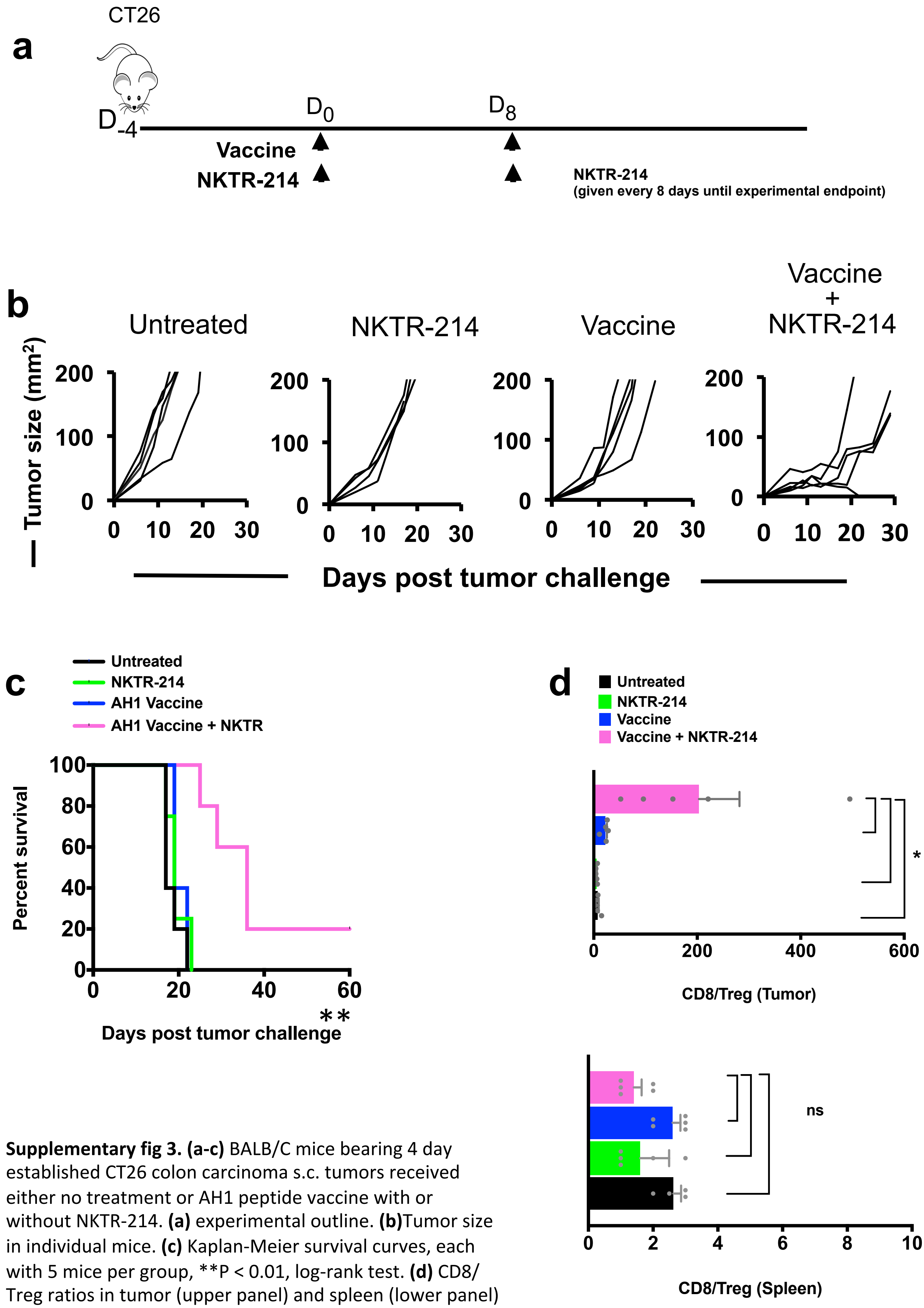

Supplementary fig 3. (a-c) BALB/C mice bearing 4 day established CT26 colon carcinoma s.c. tumors received either no treatment or AH1 peptide vaccine with or without NKTR-214. **(a)** experimental outline. **(b)**Tumor size in individual mice. **(c)** Kaplan-Meier survival curves, each with 5 mice per group, **P < 0.01, log-rank test. **(d)** CD8/ Treg ratios in tumor (upper panel) and spleen (lower panel) were analyzed on day 7 post treatment (n=5, *P<0.05, ns= non significant; one way analysis of variance).

Supplementary Fig. 4

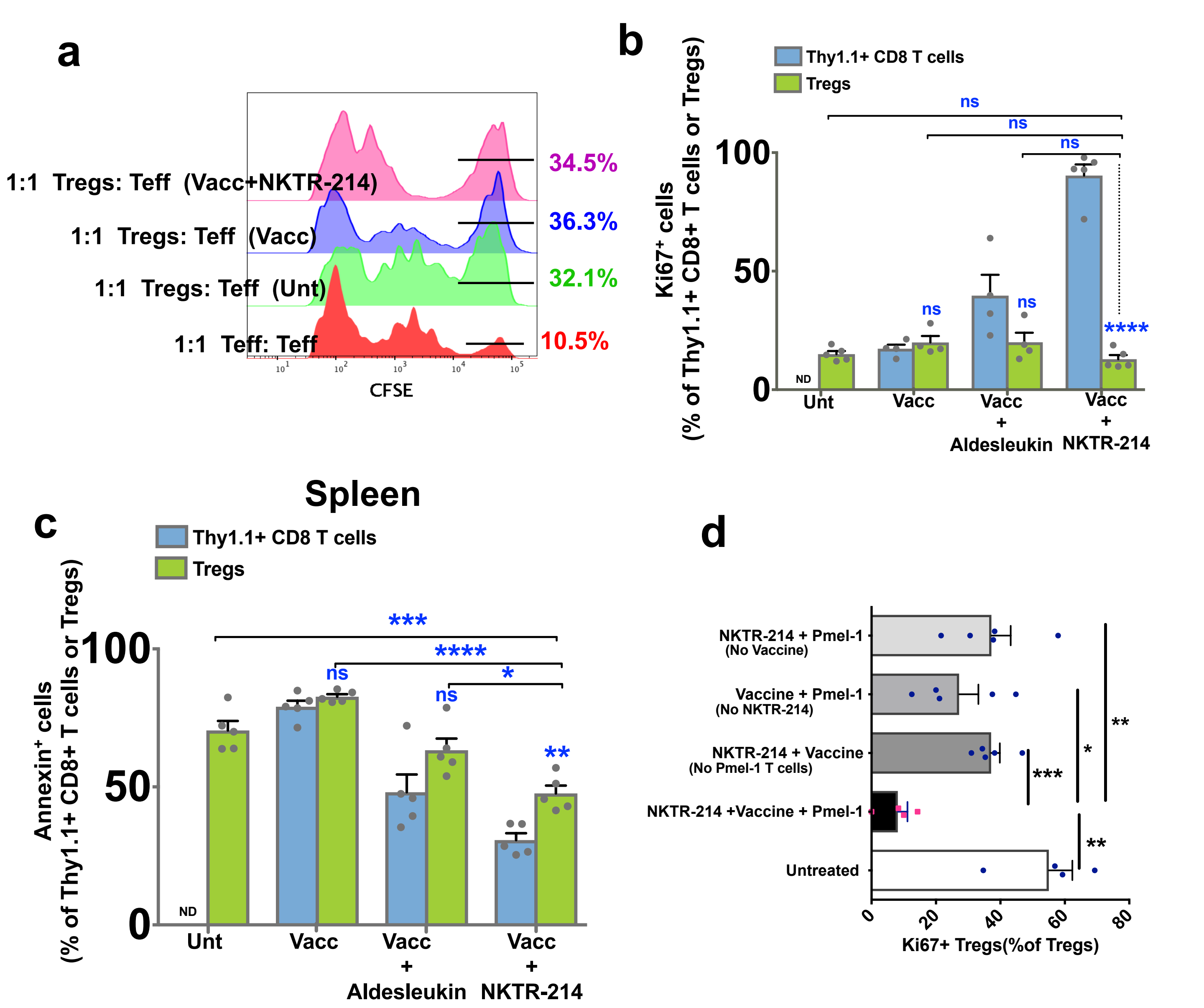

Supplementary fig 4. (a-c) C57BL/6 mice bearing 7-day established s.c. B16.F10 tumors received pmel-1 T cells and were either left untreated or received gp100 peptide vaccine with or without NKTR-214. **(a)** $CD4^+$ $CD25^-$ Teff cells and $CD4^+$ $CD25^{hi}$ Tregs were sorted on day 7 from splenocytes of either untreated mice or mice that received vaccine or vaccine plus NKTR-214. CFSE labeled $CD4^+$ $CD25^-$ responder Teff cells were cultured for 72 hours in 1:1 ratio with either unlabeled responder $CD4^+$ $CD25^-$ Teff cells or Tregs from same treatment group, in presence of anti-CD3/anti-CD28 beads and IL-2. Percentages of non-proliferated CSFE-labeled responder Teff cells are depicted in histogram. (**b-c**)Vaccinated mice either received no treatment or received aldesleukin or NKTR-214. Proliferation and apoptosis profile of pmel-1 $CD8^+$ T cells and Tregs were analyzed on day 7-post treatment in spleen of different treatment groups. (**b**) Histograms showing Ki67 and (**c**) Annexin V expression on cells. Data represented as mean± SEM, (n=4-5, *P<0.05, ***P<0.001, ****P<0.0001, ns= non significant, unpaired t-test). **(d-e)** C57BL/6 mice bearing 7-days old s.c. B16.F10 tumors, and received indicated combinations of pmel-1 T cells, gp100 peptide vaccine and NKTR-214 or anti-CD8 antibody. (d) Ki67 expression on intratumoral Tregs, (**e**) Absolute numbers of $Thy1.1^+$ $CD8^+$ Teff and Tregs analyzed on day 7 post treatment. Data is represented as mean± SEM (n = 4-5, *P<0.05, **P<0.01, ***P<0.001, unpaired t-test.).

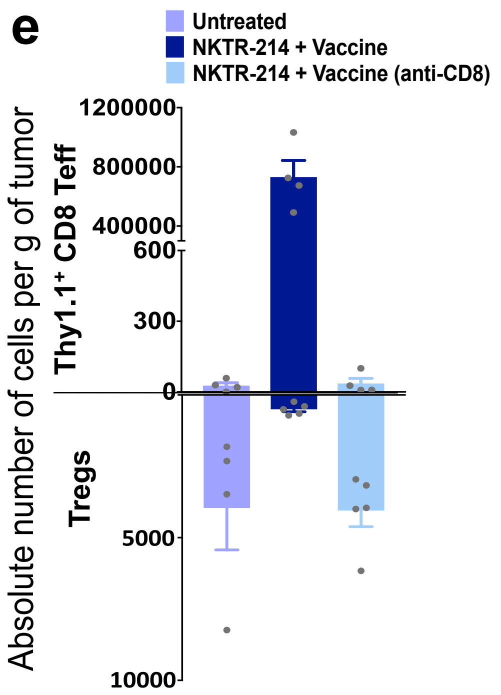

Supplementary Fig. 5

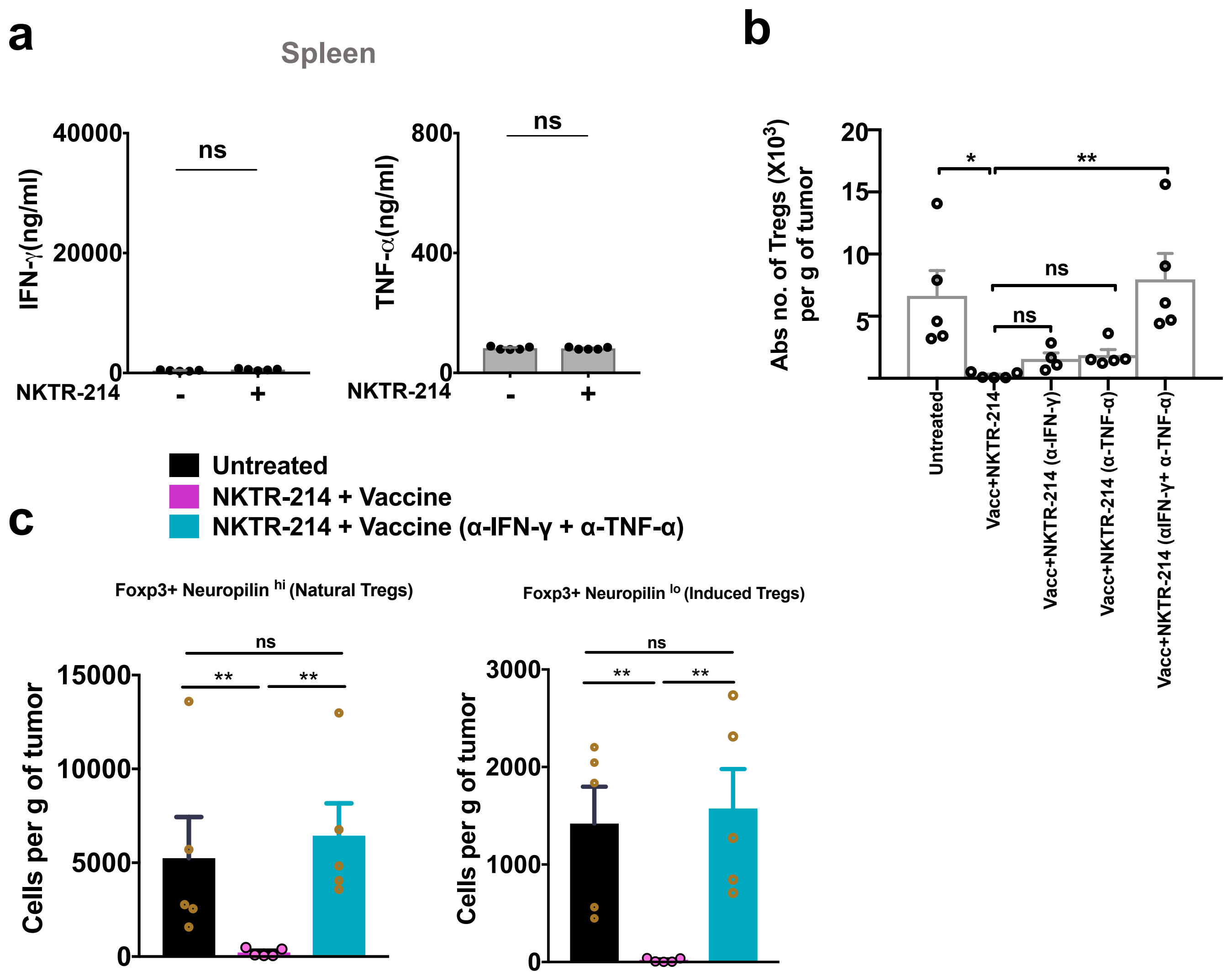

Supplementary fig 5. (**a**) C57BL/6 mice bearing 7-day established s.c. B16.F10 tumors received pmel-1 T cells and were either left untreated (-) or received NKTR-214 plus gp100 peptide vaccine (+). Level of IFN-g and TNF-a protein measured by Luminex on day 7 in spleen of untreated or vaccine-NKTR-214 treated mice. Data represented as mean±SEM (ns=non-significant, unpaired t-test). (**b-c)**) C57BL/6 mice bearing 7-day established, s.c. B16.F10 tumors either left untreated or received pmel-1 T cells followed by gp100 peptide vaccine plus NKTR-214 with indicated combinations of *in vivo* neutralizing antibodies against IFN-g and/or TNF-a given on day -1, day 2, day 4 and day 6. (**b**) Absolute numbers of Tregs (n=5, *P<0.05; **P<0.01, ns=non-significant, one way analysis of variance) and (**c**) Number of natural Tregs (upper panel) and induced Tregs (lower panel), were analyzed in tumor on day 7-post treatment. Data represented as mean±SEM. (n=5, **P<0.01, ns=non-significant, unpaired t-test)

Supplementary Fig. 6

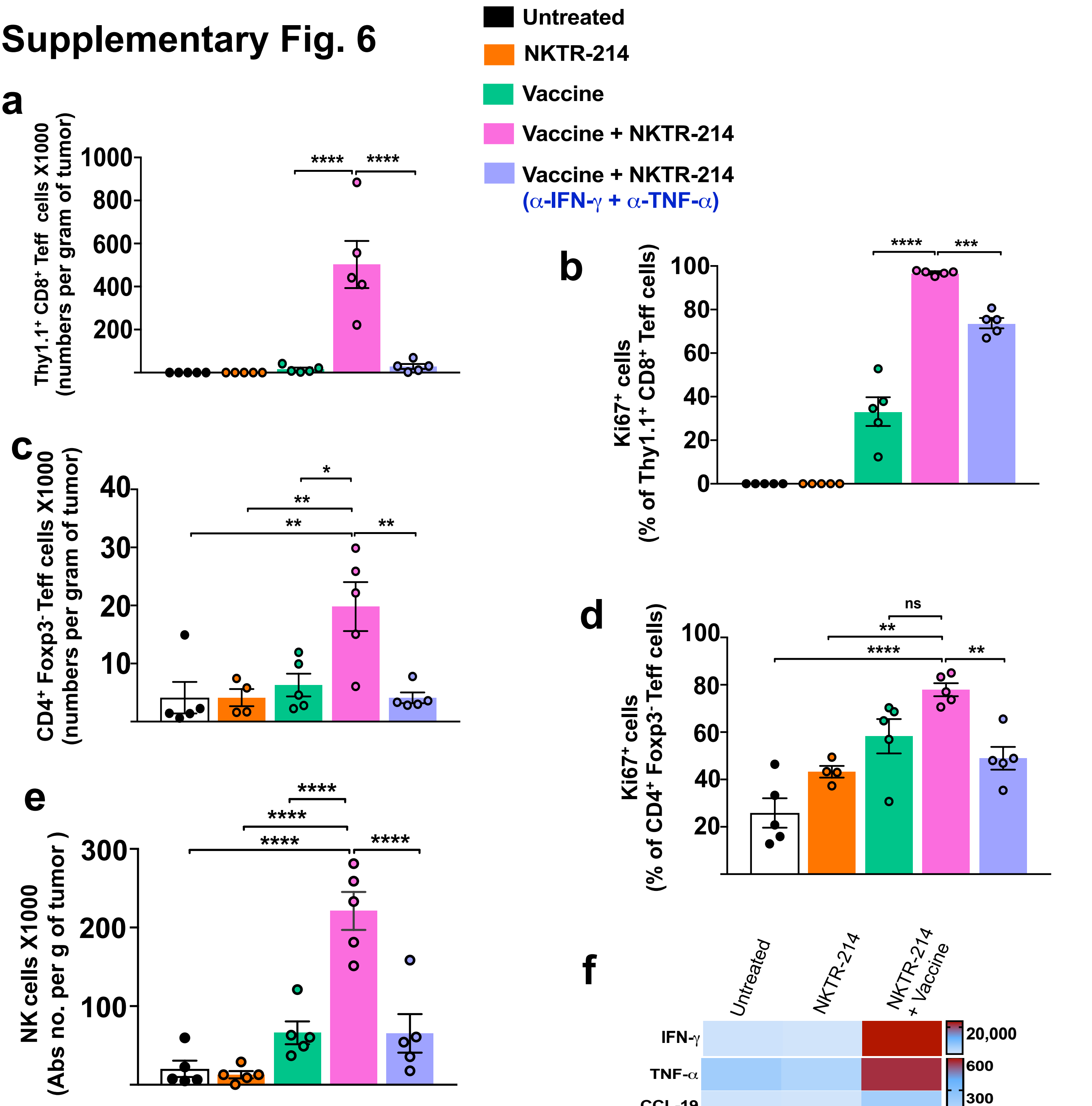

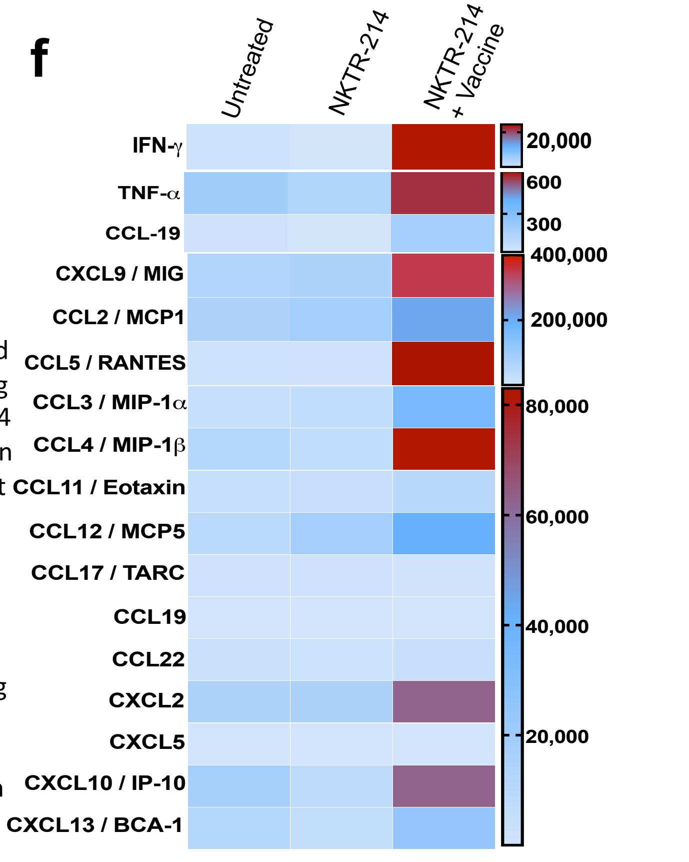

Supplementary fig 6. (**a-f**) C57BL/6 mice bearing 7-day established s.c. B16.F10 tumors received pmel-1 T cells followed by gp100 peptide vaccine plus NKTR-214 and *in vivo* neutralizing antibodies against IFN-γ and TNF-α, given on day -1, day 2, day 4 and day 6, as indicated. (**a**) Absolute numbers and (**b**) expression of Ki67 on pmel-1 CD8+ T cells in tumor on day 7-post treatment are shown. **(c)** Absolute number and **(d)** expression of Ki67 on CD4+ Foxp3- Teff in tumor on day 7 post treatment are shown. **(e)** Absolute numbers of NK cells in tumor of indicated treatment groups are shown. Data represented as mean±SEM. (n=5, *P<0.05, **P<0.01, ***P<0.001, ****P<0.0001, ns=non-significant, one way analysis of variance). (**f**) Heat map depicting protein level of various cytokines and chemokines in tumor of mice from indicated groups were analyzed 7 days after treatment. color scale denotes levels of cytokine/chemokines in pg/ml

Supplementary Fig. 7

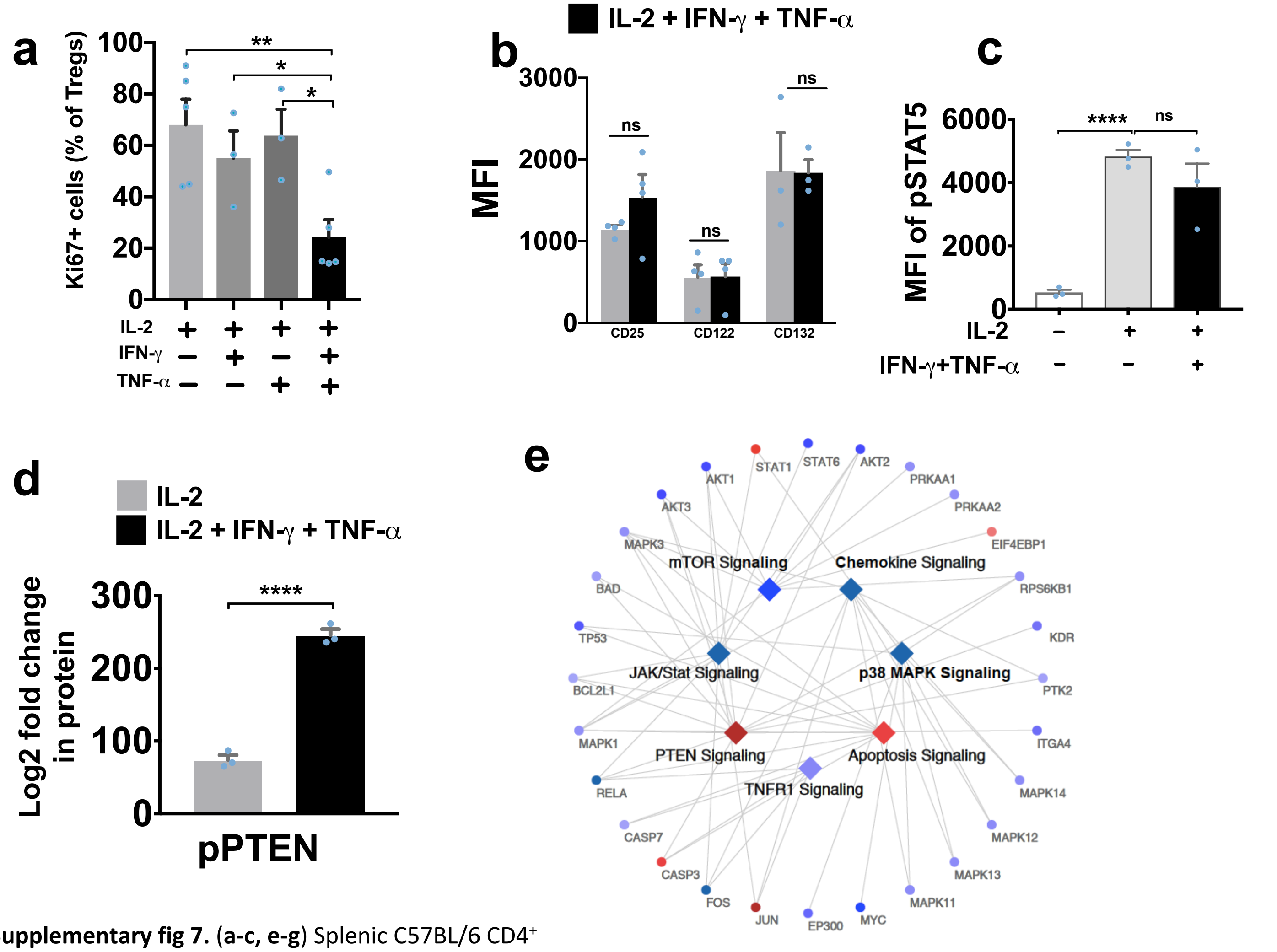

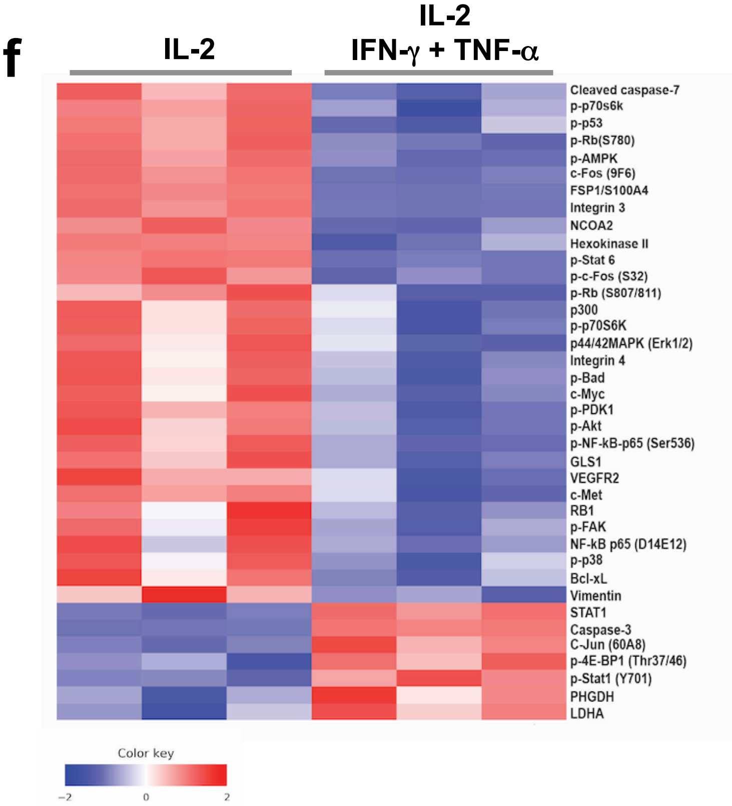

Supplementary fig 7. (**a-c, e-g**) Splenic C57BL/6 CD4+ CD25hi Tregs were cultured with anti-CD3/anti-CD28 beads and IL-2 with or without IFN-γ and/or TNF-α for 8 days and supernatant was replaced biweekly with media containing fresh cytokines. Expression of Ki67 on (**a**) Tregs; (**b**) Expression of CD25, CD122 and CD132 on Tregs on day 9 of culture. (**c**) Freshly sorted splenic Tregs were cultured with IFN-γ and TNF-α and anti-CD3/ anti-CD28 beads for 6 hours followed by addition of IL-2 for last 30 minutes of culture. Phosphorylated STAT5 expression was analyzed by flow cytometry. (**a-d**) Data (mean±SEM) is representative of three independent experiments (*P<0.05, **<0.01, ns= non-significant, unpaired t-test). (**d**) Log2 fold change in expression of PTEN analyzed by reverse phase protein array (RPPA) in protein lysates obtained from Tregs that were cultured in presence of cytokines and anti-CD3/anti-CD28 beads for 3 days followed by washing and resting overnight in complete medium. IFN-γ and TNF-α was added next day for 6 hours followed by IL-2 that was added for last 30 minutes of culture. (**e-f**) RPPA profiling was done on protein lysates obtained from cultured Tregs on day 4. (**e**) Network map depicting enriched signaling pathways and, (**f**) heat map showing differentially expressed proteins in Tregs cultured in IL-2 and IFN-γ plus TNF-α vs IL-2 alone. Data are from three experimental replicates.

Supplementary Table 1

Supplementary Table 1. Individual RPPA values and Log2 fold change in expression of differentially expressed proteins analyzed in protein lysates obtained from Tregs that were cultured in presence of TCR signaling either with IL-2 alone or with IL-2 together with IFN-γ and TNF-α.

								"IFNg+TNFa_over_IL-2 alone max. int. > 200"		
Gene_ID	Swiss_ID	IFNg+TNFa_1	IFNg+TNFa_2	IFNg+TNFa_3	IL-2 alone_1	IL-2 alone_2	IL-2 alone_3	p-value (<= 0.05)	adj_p-value	log2_FC log2(1.25), <= -log2(1.25))
JUN	P05412	2725.10	2158.86	2403.73	1212.36	1097.77	1221.97	0.0007	0.01	1.04
STAT1	P42224	39680.54	36839.86	39519.31	22367.23	21297.84	22553.76	0.0000	0.00	0.81
CASP3	P42574	3946.27	3849.84	3920.03	2366.47	2392.34	2400.77	0.0000	0.00	0.71
LDHA	P00338	423.75	342.56	390.71	252.45	190.25	282.66	0.0231	0.10	0.69
STAT1	P42224	630.76	706.00	651.28	469.75	473.22	441.12	0.0009	0.02	0.52
EIF4EBP1	Q13541	15980.40	14430.11	16331.16	11316.93	11876.42	9741.42	0.0076	0.07	0.51
PHGDH	O43175	5590.05	4736.43	5165.34	4243.01	3791.52	4278.40	0.0207	0.10	0.33
RPS6KB1	P23443	464.98	411.40	473.49	565.65	550.78	580.46	0.0078	0.07	-0.33
RB1	P06400	10200.08	8877.69	8910.11	11160.76	11607.10	12278.07	0.0133	0.09	-0.33
BCL2L1	Q07817	527.66	496.29	563.28	709.77	606.66	675.85	0.0185	0.10	-0.33
PDK1	O15530	8592.92	7604.38	8021.17	10493.75	9668.54	10155.39	0.0062	0.07	-0.33
CASP7	P55210	1173.90	1119.43	1221.77	1525.29	1407.37	1508.60	0.0028	0.04	-0.34
PTK2	Q05397	177.63	159.83	178.90	226.67	192.20	236.65	0.0321	0.12	-0.34
BAD	Q92934	810.38	699.12	769.99	1026.63	886.73	1008.14	0.0173	0.10	-0.36
MET	P08581	4163.85	3439.08	3615.16	5011.89	4758.80	4930.24	0.0099	0.08	-0.39
NCOA2	Q15596	704.91	699.67	746.54	933.02	977.31	934.63	0.0004	0.01	-0.4
MAPK1,MAPK3	P27361,P28482,P27361,P28482	3657.83	3041.11	3008.24	4477.72	3849.79	4572.87	0.0267	0.11	-0.41
RPS6KB1	P23443	2551.66	2050.43	2251.27	3184.72	2737.68	3163.27	0.0242	0.10	-0.41
PRKAA1, PRKAA2	Q13131,P54646	4079.88	3879.43	3920.13	5380.93	5067.22	5356.59	0.0003	0.01	-0.41
MAPK14,MAPK11,MAPK12,MAPK13	O15264,P53778,Q15759,Q16539	584.92	512.36	636.32	828.34	668.17	821.31	0.0374	0.14	-0.42
RB1	P06400	1431.19	1382.08	1331.69	2003.06	1872.17	2051.09	0.0005	0.01	-0.52
KDR	P35968	566.01	426.34	470.02	770.11	665.83	665.27	0.0189	0.10	-0.53
ITGA4	P13612	737.54	573.49	656.56	1037.73	823.68	1023.68	0.0215	0.10	-0.55
GLS	O94925	880.89	739.16	810.38	1240.51	1086.58	1297.45	0.0056	0.06	-0.58
EP300	Q09472	3383.07	2352.03	2695.47	4478.39	3650.61	4363.05	0.0319	0.12	-0.58
HK2	P52789	199.60	221.31	251.87	349.48	347.28	343.11	0.0029	0.04	-0.63
TP53	P04637	122.30	114.59	149.85	202.57	188.07	209.20	0.0069	0.07	-0.64
VIM	P08670	2576.31	2703.70	2212.25	3615.10	4768.99	3732.97	0.0110	0.08	-0.69
RB1	P06400	191.48	143.30	172.61	282.32	220.92	326.27	0.0267	0.11	-0.7
AKT1, AKT2, AKT3	P31749	532.35	405.64	451.54	835.56	660.91	770.03	0.0094	0.08	-0.71
STAT6	P42226	443.83	460.11	453.26	756.19	773.58	768.92	0.0000	0.00	-0.76
RELA	Q04206	2135.11	1673.43	2026.13	4198.33	2369.23	4132.04	0.0458	0.17	-0.84
MYC	P01106	1500.27	1057.26	1332.30	2726.35	1950.30	2803.51	0.0138	0.09	-0.94
RELA	Q04206	183.77	125.90	133.42	376.55	279.73	381.98	0.0053	0.06	-1.23
S100A4	P26447	853.01	788.01	904.36	5098.50	4643.06	4931.24	0.0000	0.00	-2.53
FOS	P01100	26.19	62.46	43.41	232.39	272.32	220.61	0.0025	0.04	-2.54
ITGB3	P05106	2837.66	2566.80	2670.70	16926.88	14659.35	16219.70	0.0000	0.00	-2.56
FOS	P01100	4.14	1.00	16.08	237.51	205.95	229.50	0.0075	0.07	-5.79

Auto #	DESCRIPTION	Ab ID	Company	Host	Current Validation Status	Gene Symbol	Swiss-Prot Acc
1	14-3-3 zeta, gamma, eta	1	EMD Millipore (Upstate)	Rabbit	Validated	YWHAZ	P63104
2	Akt	2	CST	Rabbit	Validated	AKT1,AKT2,AKT3	Q9Y243,P31751,P31749
3	Akt (Ser473)	3	CST	Rabbit	Validated	AKT1, AKT2, AKT3	P31749
4	Akt (Thr308)	4	CST	Rabbit	Validated	AKT1, AKT2, AKT3	P31749
5	ALK (D5F3) XP®	7	CST	Rabbit	Validated	ALK	Q9UM73
6	ALK (Tyr1586) (3B4)	8	CST	Rabbit	Validated	ALK	Q9UM73
7	ALK (Tyr1604)	9	CST	Rabbit	Validated	ALK	Q9UM73
8	AMPKα (Thr172) (40H9)	11	CST	Rabbit	Validated	PRKAA1, PRKAA2	Q13131,P54646
9	AMPKα1 (Ser485)	12	CST	Rabbit	Validated	PRKAA1	Q13131
10	AMPKβ1 (Ser108)	13	CST	Rabbit	Validated	PRKAB1	Q9Y478
11	Annexin_I	15	Invitrogen	Rabbit	Validated	ANXA1	P04083
12	ATM	18	abcam	Rabbit	Validated	ATM	Q13315
13	ATR	20	CST	Rabbit	Validated	ATR	Q13535
14	ATR (Ser428)	21	CST	Rabbit	Validated	ATR	Q13535
15	Aurora A (Thr288)/Aurora B (Thr232)/Aurora C (Thr198) (D13A11) XP®	22	CST	Rabbit	Validated	AURKA,AURKB,AURKC	Q9UQB9,Q96GD4,O14965
16	Aurora A/AIK	23	CST	Rabbit	Validated	AURKA,AURKB,AURKC	Q9UQB9,Q96GD4,O14965
17	Axl (Tyr702) (D12B2)	25	CST	Rabbit	Validated	AXL	P30530
18	Bad	26	CST	Rabbit	Validated	BAD	Q92934
19	Bad (Ser112)	27	CST	Rabbit	Validated	BAD	Q92934
20	Bad (Ser136)	28	CST	Rabbit	Validated	BAD	Q92934
21	Bad (Ser155)	29	CST	Rabbit	Validated	BAD	Q92934
22	Bak	30	CST	Rabbit	Validated	BAK1	Q16611
23	Bax	31	CST	Rabbit	Validated	BAX	Q07812
24	Bcl-2 (Ser70) (5H2)	33	CST	Rabbit	Validated	BCL2	P10415
25	Bcl-2 (Thr56) (Human Specific)	34	CST	Rabbit	Validated	BCL2	P10415
26	Bcl-xL	35	CST	Rabbit	Validated	BCL2L1	Q07817
27	BRCA1	36	EMD Millipore (Upstate)	Rabbit	Validated	BRCA1	P38398
28	BRCA2	37	CST	Rabbit	Validated	BRCA2	P51587
29	Cadherin-E (24E10)	38	CST	Rabbit	Validated	CDH1	P12830
30	Cadherin-N	39	CST	Rabbit	Validated	CDH2	P190223
31	Caspase-3	41	CST	Rabbit	Validated	CASP3	P42574
32	Phospho-Beta-Catenin (Ser33/37/Thr41)	42	CST	Rabbit	Validated	CTNNB1	P35222
33	Caveolin-1 (D46G3) XP®	43	CST	Rabbit	Validated	CAV1	Q03135
34	Caveolin-1 (Y14) (EPR2288Y)	44	Abcam-Epitomics	Rabbit	Validated	CAV1	Q03135
35	CD24 (FL-80)	45	SantaCruz	Rabbit	Validated	CD24	P25063
36	Chk1 (Ser345)	48	CST	Rabbit	Validated	CHEK1	O14757
37	Chk2 (Ser33/35)	50	CST	Rabbit	Validated	CHEK2	O96017
38	Claudin-1	52	CST	Rabbit	Validated	CLDN1	O95832
39	c-Met	54	Abcam	Rabbit	Validated	MET	P08581
40	c-Myc (phospho T58)	56	Abcam	Rabbit	Validated	MYC	P01106
41	DKK1	60	CST	Rabbit	Validated	DKK1	O94907
42	EGF Receptor (D38B1) XP®	61	CST	Rabbit	Validated	EGFR	P00533
43	EGF Receptor (L858R Mutant Specific) (43B2)	62	CST	Rabbit	Validated	EGFR	P00533
44	EGF Receptor (Ser1046/1047)	63	CST	Rabbit	Validated	EGFR	P00533
45	EGF Receptor (Tyr1045)	64	CST	Rabbit	Validated	EGFR	P00533
46	EGF Receptor (Tyr1148)	65	CST	Rabbit	Validated	EGFR	P00533
47	EGF Receptor (Tyr1173)	66	Invitrogen (BioSource)	Rabbit	Validated	EGFR	P00533
48	EGF Receptor (Tyr1173) (53A5)	67	CST	Rabbit	Validated	EGFR	P00533
49	EGF Receptor(Tyr1068) (D7A5) XP®	68	CST	Rabbit	Validated	EGFR	P00533
50	Ezh2 (D2C9) XP®	69	CST	Rabbit	Validated	EZH2	Q15910
51	FGF Receptor 1 (D8E4) XP®	70	CST	Rabbit	Validated	FGFR1	P11362
52	FoxO1 (C29H4)	72	CST	Rabbit	Validated	FOXO1	Q12778
53	FSP1/S100A4	73	EMD Millipore	Rabbit	Validated	S100A4	P26447
54	HER2/ErbB2 (Tyr1248)	77	CST	Rabbit	Validated	ERBB2	P04626
55	HER2/ErbB2 (Y877)	78	Imgenex	Rabbit	Validated	ERBB2	P04626
56	HER3/ErbB3 (1B2E)	79	CST	Rabbit	Validated	ERBB3	P21860
57	HER3/ErbB3 (Tyr1197) (C56E4)	80	CST	Rabbit	Validated	ERBB3	P21860
58	Histone Deacetylase 1 (HDAC1)	82	CST	Rabbit	Validated	HDAC1	Q13547
59	Histone Deacetylase 3 (HDAC3)	83	CST	Rabbit	Validated	HDAC3	O15379
60	Histone Deacetylase 4 (HDAC4)	84	CST	Rabbit	Validated	HDAC4	P56524
61	Histone Deacetylase 6 (HDAC6)	85	SantaCruz	Rabbit	Validated	HDAC6	Q9UBN7
62	ILK1	87	CST	Rabbit	Validated	ILK	Q13418
63	Jak1 (Tyr1022/1023)	88	CST	Rabbit	Validated	JAK1	P23458
64	Kit-c	89	Abcam	Rabbit	Validated	KIT	P10721
65	Lipocalin-1 (H-45)	91	SantaCruz	Rabbit	Validated	LCN1	P31025
66	LRP6 (C5C7)	92	CST	Rabbit	Validated	LRP6	O75581
67	MEK1	93	Abcam-Epitomics	Rabbit	Validated	MAP2K1	Q02750
68	MEK1/2 (Ser217/221)	94	CST	Rabbit	Validated	MAP2K1	Q02750
69	Met (Tyr1234/1235) (D26) XP®	95	CST	Rabbit	Validated	MET	P08581
70	MMP-9	96	CST	Rabbit	Validated	MMP9	P14780
71	mTOR	97	CST	Rabbit	Validated	MTOR	P42345
72	mTOR (Ser2448) (D9C2) XP®	98	CST	Rabbit	Validated	MTOR	P42345
73	Notch1 (C44H11)	101	CST	Rabbit	Validated	NOTCH1	P46531
74	p27 (Thr187)	105	Invitrogen(Zymed)	Rabbit	Validated	CDKN1B	P46527
75	p27 KIP1 (T198)	106	Abcam	Rabbit	Validated	CDKN1B	P46527
76	p27 KIP1(C-term)	107	Abcam-Epitomics	Rabbit	Validated	CDKN1B	P46527
77	p38 MAPK	109	CST	Rabbit	Validated	MAPK14,MAPK11,MAPK12	P53778,Q15759,Q16539

78	p38 MAPK (Thr180/Tyr182) (D3F9) XP®	111	CST	Rabbit	Validated	MAPK14,MAPK11,MAPK12,MAPK13	O15264,P53778,Q15759,Q16539
79	p44/42 MAPK (Erk1/2)	112	CST	Rabbit	Validated	MAPK1,MAPK3	P27361,P28482,P27361,P28482
80	p44/42 MAPK (Erk1/2) (Thr202/Tyr204) (197G2)	113	CST	Rabbit	Validated	MAPK1,MAPK3	P27361,P28482,P27361,P28482
81	p53	114	CST	Rabbit	Validated	TP53	P04637
82	p53 (Ser15)	115	CST	Rabbit	Validated	TP53	P04637
83	p70 S6 Kinase	116	CST	Rabbit	Validated	RPS6KB1	P23443
84	p70 S6 Kinase (Ser371)	117	CST	Rabbit	Validated	RPS6KB1	P23443
85	p70 S6 Kinase (T389)	118	CST	Rabbit	Validated	RPS6KB1	P23443
86	p70 S6 Kinase (T412)	119	EMD Millipore (Upstate)	Rabbit	Validated	RPS6KB1	P23443
87	PDGF Receptor α (Tyr754) (23B2)	121	CST	Rabbit	Validated	PDGFRA	P16234
88	PDGF Receptor β (28E1)	122	CST	Rabbit	Validated	PDGFRB	P09619
89	PDGF Receptor β (Tyr751)	124	CST	Rabbit	Validated	PDGFRB	P09619
90	PDK1 (Ser241)	125	CST	Rabbit	Validated	PDK1	Q15118
91	PIAS1 (D33A7) XP®	128	CST	Rabbit	Validated	PIAS1	O75925
92	PTEN (D4.3) XP®	129	CST	Rabbit	Validated	PTEN	P60484
93	PTEN (Ser380)	130	CST	Rabbit	Validated	PTEN	P60484
94	Raf-B (Ser445)	132	CST	Rabbit	Validated	BRAF	P15056
95	RANKL	133	Novus	Rabbit	Validated	TNFSF11	O14788
96	Ret (Tyr905)	134	CST	Rabbit	Validated	RET	P07949
97	Serpin A1(EPSISR16)	136	Abcam-Epitomics	Rabbit	Validated	SERPINA1	P01009
98	Slug (C19G7)	137	CST	Rabbit	Validated	SNAI2	O43623
99	Smad2 (Ser465/467)	140	CST	Rabbit	Validated	SMAD2	Q15796
100	SOCS1 (A156)	144	CST	Rabbit	Validated	SOCS1	O15524
101	SOCS3	145	CST	Rabbit	Validated	SOCS3	O14543
102	Sox2 (D6D9) XP®	146	CST	Rabbit	Validated	SOX2	P48431
103	Src (Tyr527)	147	CST	Rabbit	Validated	SRC	P12931
104	Stat1	149	CST	Rabbit	Validated	STAT1	P42224
105	Stat1 (Tyr701)	150	CST	Rabbit	Validated	STAT1	P42224
106	Stat2 (Tyr690)	151	CST	Rabbit	Validated	STAT2	P52630
107	Stat3 (Ser727)	153	CST	Rabbit	Validated	STAT3	P40763
108	Stat4 (Tyr693)	155	CST	Rabbit	Validated	STAT4	Q14765
109	Stat5 (Tyr694)	157	CST	Rabbit	Validated	STAT5A	P42229
110	Stat6	158	CST	Rabbit	Validated	STAT6	P42226
111	Stat6 (Tyr641)	159	CST	Rabbit	Validated	STAT6	P42226
112	Tuberin/TSC2 (Thr1462)	164	CST	Rabbit	Validated	TSC2	P49815
113	Vimentin (D21H3) XP®	166	CST	Rabbit	Validated	VIM	P08670
114	Wnt5a/b (C27E8)	167	CST	Rabbit	Validated	WNT5A	P41221
115	YAP(H125)	169	Santa Cruz	Rabbit	Validated	YAP1	P46937
116	ZO-1	170	CST	Rabbit	Validated	TJP1	Q07157
117	Caspase-7	171	CST	Rabbit	Validated	CASP7	P55210
118	VEGF Receptor2 (55B11)	173	CST	Rabbit	Validated	KDR	P35968
119	Stat5a (L-20)	174	Santa Cruz	Rabbit	Validated	STAT5A	P42229
120	SOX9*	178	EMD Millipore (Upstate)	Rabbit	Validated	SOX9	P48436
121	SAPK/JNK (T183/Y185)(81E11)	179	CST	Rabbit	Validated	MAPK8	P45983
122	ZEB1	180	Novus	Rabbit	Validated	ZEB1	P37275
123	PHF8 antibody (pAb)	185	Active Motif	Rabbit	Validated	PHF8	Q9UPP1
124	ASH2 Rabbit	198	Bethyl Laboratories	Rabbit	Validated	ASH2L	Q9UBL3
125	CBP (A-22)	200	Santa Cruz	Rabbit	Validated	CREBBP	Q92793
126	c-Fos (9F6) Rabbit mAb	201	CST	Rabbit	Validated	FOS	P01100
127	CHAF1A (D77D5) XP Rabbit mAb	202	CST	Rabbit	Validated	CHAF1A	Q13111
128	c-Jun (60A8) Rabbit mAb	203	CST	Rabbit	Validated	JUN	P05412
129	CRSP1/TRAP220	204	Bethyl Laboratories	Rabbit	Validated	MED1	Q15648
130	CtBP2 Antibody	205	CST	Rabbit	Validated	CTBP2	P56545
131	Cyclin C (T-19)	206	Santa Cruz	Rabbit	Validated	CCNC	P24863
132	DRIP130	207	AbCam	Rabbit	Validated	MED23	Q9ULK4
133	FBX011	208	AbCam	Rabbit	Validated	FBXO11	Q86XK2
134	FoxK2 Antibody	209	CST	Rabbit	Validated	FOXK2	Q01167
135	KLF4 (D1F2) Rabbit mAb	211	CST	Rabbit	Validated	KLF4	O43474
136	MED12	212	AbCam	Rabbit	Validated	MED12	Q93074
137	p300 (C-20)	215	Santa Cruz	Rabbit	Validated	EP300	Q09472
138	Phospho-c-Fos (Ser32) (D82C12) XP Rabbit mAb	216	CST	Rabbit	Validated	FOS	P01100
139	PPP1R10 [EPR11706]	217	AbCam	Rabbit	Validated	PPP1R10	Q96QC0
140	Stat3 (D3Z2G) Rabbit mAb	218	CST	Rabbit	Validated	STAT3	P40763
141	TRAP220/MED1 (phospho T1457)	220	AbCam	Rabbit	Validated	MED1	Q15648
142	HIF-2A (D9E3) Rabbit mAB	221	CST	Rabbit	Validated	EPAS1	Q99814
143	Phospho-c-Jun (Ser63) II Ab	223	CST	Rabbit	Validated	JUN	P05412
144	Sin3b	225	AbCam	Rabbit	Validated	SIN3B	O75182
145	Phospho-Rb (Ser807/811)	237	CST	Rabbit	Validated	RB1	P06400
146	Phospho-Rb (Ser780) (C84F6)	238	CST	Rabbit	Validated	RB1	P06400
147	Phospho-FAK (Tyr576/577)	261	CST	Rabbit	Validated	PTK2	Q05397
148	Ki67	265	Vector Laboratories	Rabbit	Validated	MKI67	P46013
149	Integrin α4 (D2E1) XP® Rabbit mAb	266	CST	Rabbit	Validated	ITGA4	P13612
150	Integrin α5 Antibody	267	CST	Rabbit	Validated	ITGA5	P08648
151	Integrin αV Antibody	268	CST	Rabbit	Validated	ITGAV	P06756
152	Integrin β1 (D2E5) Rabbit mAb	269	CST	Rabbit	Validated	ITGB1	P05556
153	Integrin β3 (D7X3P) XP® Rabbit mAb	270	CST	Rabbit	Validated	ITGB3	P05106
154	Integrin β4 Antibody	271	CST	Rabbit	Validated	ITGB4	P16144
155	PI3 Kinase p85 Antibody	286	CST	Rabbit	Validated	PIK3R1,PIK3R2	P27986, O00459
156	AMPKa (23A3) Rabbit mAb	287	CST	Rabbit	Validated	PRKAA1	Q13131
157	PI3 Kinase p110α (C73F8) Rabbit mAb	288	CST	Rabbit	Validated	PIK3CA	P42336

158	Estrogen Receptor (SP1)	295	Thermo	Rabbit	Validated	ESR1	P03372
159	MEK6	300	Abcam	Rabbit	Validated	MAP2K6	P525643
160	p21 Waf1/Cip1 (12D1)	301	CST	Rabbit	Validated	CDKN1A	P38936
161	Atg12 (D88H11) Rabbit mAb	304	CST	Rabbit	Validated	ATG12	O94817
162	Beclin-1 (D40C5) Rabbit mAb	305	CST	Rabbit	Validated	BECN1	Q14457
163	Cleaved Caspase-3 (Asp175) Antibody	306	CST	Rabbit	Validated	CASP3	P42574
164	Beta-Catenin Rabbit (CT)	307	Invitrogen	Rabbit	Validated	CTNNB1	P35222
165	Atg3	312	CST	Rabbit	Validated	ATG3	Q9NT62
166	Atg7	314	CST	Rabbit	Validated	ATG7	O95352
167	LC3A (D50G8) XP	315	CST	Rabbit	Validated	MAP1LC3A	Q9H492
168	LC3B (D11) XP	316	CST	Rabbit	Validated	MAP1LC3B	Q9GZQ8
169	EGF Receptor (Tyr845)	318	CST	Rabbit	Validated	EGFR	P00533
170	EGF Receptor (Tyr992)	319	CST	Rabbit	Validated	EGFR	P00533
171	ErbB2/HER2 (Y1248)	322	Imgenex	Rabbit	Validated	ERBB2	P04626
172	HER2/c-ErbB2	323	Dako	Rabbit	Validated	ERBB2	P04626
173	Phospho-Shc (Tyr317) Antibody	325	CST	Rabbit	Validated	SHC1	P29353
174	Steroid-5-alpha-reductase 1 (SRD5A1)	326	Novusbio	Rabbit	Validated	SRD5A1	P18405
175	Fatty Acid Synthase Antibody	328	CST	Rabbit	Validated	FAS	P25445
176	Hexokinase II (C64G5) Rabbit mAb	329	CST	Rabbit	Validated	HK2	P52789
177	Anti-PFKFB3 antibody-C-terminal	331	Abcam	Rabbit	Validated	PFKFB1,PFKFB2,PFKFB3,PFKFB4	P16118,O60825,Q16875,Q16877
178	Anti-RRM2 antibody [EPR11820]	332	Abcam	Rabbit	Validated	RRM2	P31350
179	GLDC	337	CST	Rabbit	Validated	GLDC	P23378
180	KGA-Specific GLS1	338	Proteintech	Rabbit	Validated	GLS	O94925
181	LDHA	339	CST	Rabbit	Validated	LDHA	P00338
182	PHGDH	340	CST	Rabbit	Validated	PHGDH	O43175
183	PKM2	341	Bethyl	Rabbit	Validated	PKM	P14618
184	PKM1/2 (C103A3)	342	CST	Rabbit	Validated	PKM	P14618
185	Aldehyde Dehydrogenase (ALDH)	5	BD Biosciences	Mouse	Validated	ALDH1A1	P00352
186	Aldehyde Dehydrogenase 2 (ALDH2)	6	Abcam	Mouse	Validated	ALDH2	P05091
187	ATM (Ser1981) (10H11.E12)	19	CST	Mouse	Validated	ATM	Q13315
188	Axl	24	Abcam	Mouse	Validated	AXL	P30530
189	CD24 (GPI-linked surface mucin) Ab-2 (SN3b)	46	Thermo	Mouse	Validated	CD24	P25063
190	Chk2 (1C12)	49	CST	Mouse	Validated	CHEK2	O96017
191	Cox-2	57	BD Biosciences	Mouse	Validated	MT-CO2	P00403
192	GATA3	74	BD Biosciences	Mouse	Validated	GATA3	P23771
193	GSK-3α/ß(0011-A)	75	Santa Cruz	Mouse	Validated	GATA3	P23771
194	HER2/c-ErbB2 P185 (e2-4001)	76	Invitrogen (BioSource)	Mouse	Validated	ERBB2	P04626
195	HIF-1α (54)	81	BD Biosciences	Mouse	Validated	HIF1A	Q16665
196	IkappaB-alpha (S32/36) (39A1431)	86	BD Biosciences	Mouse	Validated	NFKBIA	P25963
197	S100A7 calcium binding protein	135	Abnova	Mouse	Validated	S100A7	P31151
198	TWIST (Twist2C1a)	165	SantaCruz	Mouse	Validated	TWIST1	Q15672
199	SRC-3 clone 1208/D1	190	BCM-Mab	Mouse	Validated	NCOA3	Q9Y6Q9
200	c-myc clone # 1123	191	BCM-Mab	Mouse	Validated	MYC	P01106
201	SRC-2 (TIF2)	192	BD	Mouse	Validated	NCOA2	Q15596
202	SRC-1 (clone 1135/H4, 1136/H4H6)	193	BCM-Mab	Mouse	Validated	NCOA1	Q15788
203	Total PR(MouseMab 1294)	194	Celetta	Mouse	Validated	PGR	P06401
204	Rb(4H1)	236	CST	Mouse	Validated	RB1	P49841
205	Mouse Anti-Human FAK (pY397)	262	BD Biosciences	Mouse	Validated	PTK2	Q05397
206	AR441 Mab	264	BCM	Mouse	Validated	AR	P10275
207	AMPKα (F6) Mouse mAb	285	CST	Mouse	Validated	PRKAA1	Q13131
208	BCL2 Oncoprotein Clone 124	296	Dako	Mouse	Validated	BCL2	P10415
209	c-Src (B-12)	308	Santa Cruz	Mouse	Validated	FYN,SRC,YES1	P06241,P12931,P07947
210	IGF-IR (3B7)	309	Santa Cruz	Mouse	Validated	IGF1R	P08069
211	Laminin-5 (gamma2 chain), clone D4B5	311	EMD Millipore	Mouse	Validated	LAMC2	Q13753
212	Aromatase-A Clone 677H7F10	320	BCM-Mab	Mouse	Validated	CYP19A1	P11511
213	Monoclonal Anti-AOX1, clone AO15	327	Sigma-Aldrich	Mouse	Validated	AOX1	Q06278
214	Anti-IDH2	330	Abcam	Mouse	Validated	IDH2	P48735
215	Anti-SCD1 antibody [CD.E10]	333	Abcam	Mouse	Validated	SCD	O00767
216	DNA-PKCS (C-19)	187	Santa Cruz	Goat	Validated	PRKDC	P78527
