# Supplementary material for: Bempegaldesleukin selectively depletes intratumoral Tregs and potentiates T cell-mediated cancer therapy
Source: Nat Commun. 2020 Jan 31;11:661. doi: 10.1038/s41467-020-14471-1 (PMC6994577; doi:10.1038/s41467-020-14471-1)
Supplement: Supplementary file 2 — Reporting Summary [file 41467_2020_14471_MOESM2_ESM.pdf]

nature research

Corresponding author(s): Willem W. Overwijk

Last updated by author(s): Dec 20, 2019

Reporting Summary

Nature Research wishes to improve the reproducibility of the work that we publish. This form provides structure for consistency and transparency in reporting. For further information on Nature Research policies, see Authors & Referees and the Editorial Policy Checklist .

Statistics

For all statistical analyses, confirm that the following items are present in the figure legend, table legend, main text, or Methods section.

n/a	Confirmed	
☐	☒	The exact sample size (*n*) for each experimental group/condition, given as a discrete number and unit of measurement
☐	☒	A statement on whether measurements were taken from distinct samples or whether the same sample was measured repeatedly
☐	☒	The statistical test(s) used AND whether they are one- or two-sided<br>*Only common tests should be described solely by name; describe more complex techniques in the Methods section.*
☒	☐	A description of all covariates tested
☒	☐	A description of any assumptions or corrections, such as tests of normality and adjustment for multiple comparisons
☐	☒	A full description of the statistical parameters including central tendency (e.g. means) or other basic estimates (e.g. regression coefficient) AND variation (e.g. standard deviation) or associated estimates of uncertainty (e.g. confidence intervals)
☒	☐	For null hypothesis testing, the test statistic (e.g. *F*, *t*, *r*) with confidence intervals, effect sizes, degrees of freedom and *P* value noted<br>*Give P values as exact values whenever suitable.*
☒	☐	For Bayesian analysis, information on the choice of priors and Markov chain Monte Carlo settings
☒	☐	For hierarchical and complex designs, identification of the appropriate level for tests and full reporting of outcomes
☒	☐	Estimates of effect sizes (e.g. Cohen's *d*, Pearson's *r*), indicating how they were calculated

Our web collection on statistics for biologists contains articles on many of the points above.

Software and code

Policy information about availability of computer code

Data collection	Flow cytometry data was acquired with FACS diva software version 6.0 and 8.0 (BD Biosciences, CA).
Data analysis	Flow cytometry data analysis was done using Flow jo v10 (Tree Star, Ashland, OR) , histology and immunofluorescence staining was analyzed using Leica Application Suite X software (Leica Microsystems Inc, IL). For RPPA analysis, fluorescence-labeled slides were scanned on a GenePix 4400 AL scanner and images were analyzed with GenePix Pro 7.0 (Molecular Devices). Gene expression was analyzed using Human PanCancer Immune Profiling Panel followed by analysis using nSolver software 4.0 (NanoString Technologies). Multiplex cytokine analysis of Luminex data and tumor size measurement were analyzed in excel software (Microsoft). RPPA data was analyzed using microsoft excel, Inguinity pathway analysis (Qiagen Bioinformatics) and Cytoscape (online bioinformatics software platform). GraphPad Prism version 6.0, 7.0 and 8.0 was used for data analysis, statistics and presentation.

For manuscripts utilizing custom algorithms or software that are central to the research but not yet described in published literature, software must be made available to editors/reviewers. We strongly encourage code deposition in a community repository (e.g. GitHub). See the Nature Research guidelines for submitting code & software for further information.

Data

Policy information about availability of data

All manuscripts must include a data availability statement. This statement should provide the following information, where applicable:
- Accession codes, unique identifiers, or web links for publicly available datasets
- A list of figures that have associated raw data
- A description of any restrictions on data availability

The raw data underlying main and supplementary figures of this study are provided as source data file. The source data for Fig. 7 d and e, supplementary Fig.7e and f are provided in supplementary Table 1.

Field-specific reporting

Please select the one below that is the best fit for your research. If you are not sure, read the appropriate sections before making your selection.

☒ Life sciences ☐ Behavioural & social sciences ☐ Ecological, evolutionary & environmental sciences

For a reference copy of the document with all sections, see nature.com/documents/nr-reporting-summary-flat.pdf

Life sciences study design

All studies must disclose on these points even when the disclosure is negative.

Sample size	To determine sample size required for each group, web-based excel sheet calculator was used (https://www.bu.edu/researchsupport/compliance/animal-care/working-with-animals/research/sample-size-calculations-iacuc/). The calculation indicated that in order to achieve more than 80% power of analysis and to see significant differences (p<0.05) between control and treatment groups, the sample size in each group should be 4-5. 4-10 (>3) samples were used in all murine experiments. After tumor induction, mice were randomized before treatment. All in vitro studies were separately repeated at least three times to ensure reproducibility. RPPA analysis was done on three experimental and three technical replicates of each sample.
Data exclusions	No data was excluded from analysis.
Replication	All in vivo murine tumor experiments were successfully replicated at least twice with separate experiments.
Randomization	After tumor induction, mice were randomized before treatment.
Blinding	Since, mice were already randomized to maintain same average tumor size before treatment in all the experimental groups, investigators were not blinded to group allocation.

Reporting for specific materials, systems and methods

We require information from authors about some types of materials, experimental systems and methods used in many studies. Here, indicate whether each material, system or method listed is relevant to your study. If you are not sure if a list item applies to your research, read the appropriate section before selecting a response.

Materials & experimental systems

n/a	Involved in the study	
☐	☒	Antibodies
☐	☒	Eukaryotic cell lines
☒	☐	Palaeontology
☐	☒	Animals and other organisms
☐	☒	Human research participants
☐	☒	Clinical data

Methods

n/a	Involved in the study	
☒	☐	ChIP-seq
☐	☒	Flow cytometry
☒	☐	MRI-based neuroimaging

Antibodies

Antibodies used	Anti-IFN-γ (Clone XMG1.2, Cat no. BE0055); anti-TNF-α (Clone XT3.11, Cat no. BP0058); anti-CTLA-4 (clone 4F10, Cat no.BE0032) and anti-PD-1 (clone RMP1-14, Cat no. BE0146); all were from BioXcell.<br>For mice immune analysis by flow cytometry, following antibodies were used. Ki67 (Clone B56, Cat no. 556027),CD3e (clone 17A2, Cat no. 740268), CD25 (clone : PC61; Cat.no. 552880), Anti-STAT5 (clone pY694, Cat No. 612599) from BD Biosciences. CD8 (clone 53-6.7, Cat no. 100733), CD4 (clone GK1.5, Cat no. 100430), CD122 (Clone TM-β1, Cat no. 123212), CD132 (clone TVGm2, Cat no. 132307) from Biolegend. CD90.1 (Clone HIS51, Cat no. 48-0900-82) and Foxp3 (clone FJK-16s, Cat no. 12-5773-82) from eBiosciences (Thermo Fisher). Anti-CD40 (Clone FGK4.5/FGK45, Cat no. BE0016-2, BioXcell, NH). Anti-CD8 (clone YTS 169.4, BE0117, BioXcell, NH). Rabbit monoclonal anti-gp100 (clone EP4863(2), Cat no. ab137078, Abcam). Goat anti-rabbit Alexa 488 (Cat no. A-11034, Invitrogen. ThermoFisher Scientific).Anti-mouse CD304(neuropilin)(Clone 3DS304M, Cat no. 12-3041-82, Invitrogen, Thermo Fisher Scientific).<br><br>For human immune analysis, following antibodies were used. CD3 (clone SK7, Cat no. 345764), CD4 (clone RPA-T4; Cat. no. 560158), CD8 (clone RPA-T8, Cat. no. 565695) were from BD Biosciences and Foxp3 eFluor 450 (clone PCH101, Cat no. 48-4776-42) from eBiosciences (Thermo Fisher).
Validation	Anti IFN-γ (Application:in vivo IFNγ neutralization, in vitro IFNγ neutralization, ELISPOT, Flow cytometry, Western blot; RRID AB_1107694; Citations: PMID: 28815056).<br>Anti-TNF-α (Application: in vivo TNFα neutralization, in vitro TNFα neutralization, Western blot; RRID AB_1107764; Citations-PMID: 27677358)

Anti-CTLA-4 (Application: in vivo CTLA-4 neutralization, in vitro CTLA-4 neutralization, Flow cytometry, Western blot; RRID AB_1107598; Citation- PMID: 28321130)
Anti-PD-1 (Application: in vivo blocking of PD-1/PD-L signaling; RRID- AB_10949053; Citations- PMID: 29038298, PMID: 29245981).
Anti-mouse CD90.1 (Application: Flow cytometry; Species reactivity: Mouse and rat; RRID: AB_1272254; Citations: PMID: 28363952 and PMID: 29888735).
Ki67 (Application: intracellular staining flow cytometry; reactivity: human, mouse, rat; PMID: 1831764).
Anti-mouse CD3 (Application: Flow cytometry; Species reactivity : Mouse; PMID: 1358260)
Anti-mouse CD25(Application: Flow cytometry; reactivity: mouse, PMID: 10899916).
Anti-STAT5 (Application: intracellular staining flow cytometry; Species reactivity: human, mouse, rat; PMID: 22271576).
Anti-mouse CD8 (Application: flow cytometry; reactivity: mouse; RRID: AB_2075239; PMID: 23460738).
Anti-mouse CD4 (Application: flow cytometry; reactivity: mouse; RRID: AB_493699; PMID: 23529616).
Anti-mouse CD122 (Application: flow cytometry; reactivity: mouse; RRID: AB_2562539; PMID: 1918958).
Anti-mouse CD132 (Application: flow cytometry; reactivity: mouse, RRID: AB_10643575 ; PMID: 30380412).
Foxp3 (Application: Flow Cytometry, Immunofluorescence, Immunohistochemistry,Immunocytochemistry, Functional Assay, Western Blot, In vitro Assay, Immunohistochemistry, Neutralization; reactivity: Bovine, Dog, Cat, Mouse, Pig, Rat; RRID: AB_465936; PMID: 28197366).

Anti-human CD3 (Application: flow cytometry; reactivity: human; PMID: 3278747).
Anti-human CD4 (Application: flow cytometry; reactivity: human; PMID: 8800551).
Anti-human CD8 (Application: flow cytometry; reactivity Human, Rhesus, Cynomolgus, Baboon; PMID: 10587353).
Anti-human Foxp3 (Application: flow cytometry; reactivity: human. non-human primates; RRID:AB_1834364, PMID:PMID: 29576375).

Eukaryotic cell lines

Policy information about cell lines

Cell line source(s)	LLC, EMT6, B16.F10 and CT26 cell lines were from ATCC. Pan02, MBT2, H22, BR5FVB cell lines were from Crown Biosciences.
Authentication	None of the cell lines used were authenticated.
Mycoplasma contamination	Cell lines were negative for mycoplasma contamination.
Commonly misidentified lines (See ICLAC register)	No commonly misidentified lines were used in the study.

Animals and other organisms

Policy information about studies involving animals; ARRIVE guidelines recommended for reporting animal research

Laboratory animals	Six-to-eight weeks old female C57BL/6 or BALB/c mice were purchased from Charles River Laboratory. Pmel-1 TCR transgenic mice on a C57BL/6 background (The Jackson Laboratory, Bar Harbor, ME) were crossed with CD90.1 congenic mice to yield pmel-1+/+ × CD90.1+/+ mice, referred to as pmel-1 mice. C3H strain and FVB strain mouse strains were purchased from The Jackson laboratory.
Wild animals	No wild animals were used in the study.
Field-collected samples	No Field-collected samples used in the study.
Ethics oversight	Animal experiments performed in this study were approved by the Institutional Animal Care and Use Committee (IACUC) of the University of Texas MD Anderson Cancer Center.

Note that full information on the approval of the study protocol must also be provided in the manuscript.

Human research participants

Policy information about studies involving human research participants

Population characteristics	All the patients were of >18 years of age. 66.6% of patients were male and 33.3% were female. Average age of male patients were 58.1 years and for female were 60.4 years. 33% of patients were diagnosed with Melanoma while 67% were detected with Renal cell carcinoma (RCC).<br>13.3 % received NKTR-214 q21d at dosing 0.003 mg/kg<br>60 % received NKTR-214 q21d at dosing 0.006 mg/kg

	26.6 % received NKTR-214 q21d at dosing 0.009 mg/kg
Recruitment	Eligible patients were treatment-refractory to ≥1 prior therapy for advanced or locally-advanced disease. Patients with a histologically confirmed diagnosis of RCC and melanoma not amenable to curative therapy were enrolled. All patients provided written informed consent before any protocol-specified procedures. Melanoma or renal cell carcinoma patients were recruited, received treatment with 0.003 or 0.006 or 0.009 mg per kg body weight (mg/kg) of NKTR-214 every 3 weeks (q21d).
Ethics oversight	MD Anderson Cancer Center, Houston, TX

Note that full information on the approval of the study protocol must also be provided in the manuscript.

Clinical data

Policy information about clinical studies

All manuscripts should comply with the ICMJEguidelines for publication of clinical research and a completedCONSORT checklist must be included with all submissions.

Clinical trial registration	NCT02869295
Study protocol	The full study protocol is detailed in manuscript.
Data collection	Melanoma and renal cell carcinoma patients were treated with NKTR-214 0.003 or 0.006 or 0.009 mg per kg of body weight, every 21 days (q21d). Blood and tumor biopsies were obtained on the first day of treatment and after 3 weeks post NKTR-214 treatment. Patient samples were collected at MD Anderson Cancer Center, Houston, TX, Yale new Haven Hospital, CTand Smilow Cancer Hospital Care Center at Yale, New Haven, CT
Outcomes	Primary Outcome Measures :<br>1. Safety of NKTR-214 as evaluated by incidence of drug-related adverse events (AEs), serious adverse events (SAEs), adverse events leading to discontinuation, deaths and clinical laboratory test abnormalities [Time Frame: 30 days after last dose]<br><br>2. Tolerability of NKTR-214 as evaluated by incidence of dose limiting toxicities (DLTs), drug-related adverse events (AEs), serious adverse events (SAEs), adverse events leading to discontinuation, deaths and clinical laboratory test abnormalities [Time Frame: 30 days after last dose]<br><br>Secondary Outcome Measures :<br><br>1. Objective response rate (ORR) of NKTR-214 based on investigator review of radiographic images [Time Frame: Through study completion, an expected average of 1 year]<br><br>2. Best overall response (BOR) in the population of interest [Time Frame: Through study completion, an expected average of 1 year]<br><br>3. Duration of Response (DOR) [Time Frame: Through study completion, an expected average of 1 year]<br>It is defined as time between the date of first radiographic documented objective response and the date of the radiographic documented disease progression.<br><br>4. Progression-Free Survival (PFS) [Time Frame: Through study completion, an expected average of 1 year]<br>PFS is defined as the time from date of enrollment to the date of the first objectively documented tumor progression or death due to any cause<br><br>5. Clinical benefit rate (CBR) [Time Frame: Through study completion, an expected average of 1 year]<br>Clinical benefit rate will be assessed as the number of subjects with confirmed complete response (CR), partial response (PR), or stable disease (SD) (where the duration of SD should be ≥ 3 months) divided by the total number of subjects in the Response Evaluable Population<br><br>6. Median time to response (MTR) [Time Frame: Through study completion, an expected average of 1 year]<br>The median time to response will be summarized descriptively for subjects who have a CR or PR.<br><br>7. Overall Survival (OS) [Time Frame: Within 3 years from study start]<br>Overall survival is defined as the time from date of enrollment to the date of death.<br><br>8. Maximum observed plasma concentration (Cmax) of NKTR-214 [Time Frame: Day 1 of Cycle 1 and 2: pre-dose and end of administration, 0.5 hr., 3 hr., 6 hr., Post dosing on Days 2, 3, 4, 5, 8, 11, and 15 for Cycle 1 and 2]<br><br>9. Time of maximum observed plasma concentration (Tmax) of NKTR-214 [Time Frame: Day 1 of Cycle 1 and 2: pre-dose and end of administration, 0.5 hr., 3 hr., 6 hr., Post dosing on Days 2, 3, 4, 5, 8, 11, and 15 for Cycle 1 and 2]<br><br>10. Area under the plasma concentration time curve in the dosing interval AUC(TAU) of NKTR-214 [Time Frame: Day 1 of Cycle 1

and 2: pre-dose and end of administration, 0.5 hr., 3 hr., 6 hr., Post dosing on Days 2, 3, 4, 5, 8, 11, and 15 for Cycle 1 and 2]

11. Half life (t½) of NKTR-214 [Time Frame: Day 1 of Cycle 1 and 2: pre-dose and end of administration, 0.5 hr., 3 hr., 6 hr., Post dosing on Days 2, 3, 4, 5, 8, 11, and 15 for Cycle 1 and 2]

12. Functional and phenotypic characterization of peripheral immune cells by flow cytometry [Time Frame: Day 1 and Day 8 of Cycle 1 and 2]

13. Changes in soluble cytokines and chemokines by multiplex immunoassay [Time Frame: Day 1 and Day 8 of Cycle 1 and 2]

14. Functional and phenotypic characterization of tumor immune infiltrates (TIL) by flow cytometry. [Time Frame: Pre-dose and week 3 after first dose]

15. Functional and phenotypic characterization of tumor immune infiltrate (TIL) by next generation sequencing of T cell receptors [Time Frame: Pre-dose and week 3 after first dose]

16. Functional and phenotypic characterization of tumor immune infiltrate (TIL) by immunohistochemistry (IHC) [Time Frame: Pre-dose and week 3 after first dose]

17. Immunogenicity analysis to assess antibodies to NKTR-214 in human serum [Time Frame: Screening and pre-dose of Day 1 of Cycle 2 and odd-numbered cycles there after (Cycle 3, 5, 7...)]

Flow Cytometry

Plots

Confirm that:

- [x] The axis labels state the marker and fluorochrome used (e.g. CD4-FITC).
- [x] The axis scales are clearly visible. Include numbers along axes only for bottom left plot of group (a 'group' is an analysis of identical markers).
- [x] All plots are contour plots with outliers or pseudocolor plots.
- [x] A numerical value for number of cells or percentage (with statistics) is provided.

Methodology

Sample preparation

For analysis in mice, single cell suspensions from spleen and tumor were prepared in PBS with 10% FCS, 2 Mm EDTA (Sigma-Aldrich, Missouri) by mashing tissue against surface of 40µm cell strainer using a plunger of 3ml syringe (BD biosciences, CA). Lymphocytes from tumor tissue were enriched on a Ficoll gradient (Histopaque 1119) while RBCs (red blood cells) were removed from spleen samples using a hypotonic lysis buffer (StemCell Technologies, MA). Blood from mice was drawn by tail snipping method and RBCs were lysed using ACK lysis buffer (Thermo Fisher Scientific, MA), to get peripheral blood mononuclear cells (PBMCs) suspension. Cells obtained from blood, tumor and spleen, were then blocked with 10% rat serum in 1XPBS and stained LIVE/DEAD TM fixable aqua dead cell stain followed by fluorochrome-conjugated antibodies. Cell were either acquired and analyzed or were fixed and permeabilized using eBiosciences Foxp3 staining kit following manufacturer's protocol. Cells were then stained for intracellular markers. Phosphorylated (p)STAT5 staining was done using transcription phosphor buffer set (BD Biosciences, CA) following manufacturer’s protocol. Acquisition was performed on a BD Fortessa flow cytometer followed by data analysis with FlowJo software.
For human sample analysis, whole blood was collected and processed to obtain peripheral blood mononuclear cells (PBMCs) while tumor biopsies were disaggregated using the BD Medimachine system according to manufacturer’s instructions. Suspension cells from either blood or tumor were stained with fluorochrome-conjugated, anti-human antibodies CD3 FITC (SK7), CD4 ACP-H7 (RPA-H4), CD8 PB (RPA-T8); all antibodies were from BD and Foxp3 eFluor 450 (PCH101), from eBiosciences, San Diego, CA. Intracellular Foxp3 and cell-surface CD25hi were used to discriminate between conventional CD4+ T cells and CD4+ Treg cells. Live/DEAD Fixable Aqua Dead Cell Stain Kit (Invitrogen Carlsbad, CA) was used to discard dead cells. Briefly, cells were incubated with antibodies for 30 minutes, then washed and fixed with paraformaldehyde 1% (Sigma). Intracellular staining for Foxp3 was performed using Foxp3 Fixation Kit (eBioscience, Thermo Fisher, MA). Stained cells were acquired on LSRII Fortessa and Canto II ((BD Biosciences, CA).

Instrument

Acquisition was performed on Fortessa, Fortessa X-20, LSR-II and Canto-II flow cytometers (BD Biosciences). Sorting was done on Aria I or II (BD Biosciences).

Software

Flow cytometry data was collected using FACS Diva (BD Biosciences) software and analyzed by using Flow jo v10 software.

Cell population abundance

Post sort, Tregs (CD4+ CD25hi) were 1-2 % and CD4+ Teff (CD4+ CD25-) were in a range of 15-20% of parent population (live single cell CD4+ T cell). Purity of Tregs were tested based on Foxp3 expression (>96%).

Gating strategy

Lymphocytes cells were gated in FCS and SSC plot, single cells were selected by selected population on a SSH (x-axis)-SSC-A (y-axis) and FSC-H (x-axis)-FSC-A (y-axis) plot. Dead cells were excluded by selecting cell population that was negative for Live/dead fixable dye aqua. Live cells were then plotted with other surface or intracellular markers for analysis or sorting.

☒ Tick this box to confirm that a figure exemplifying the gating strategy is provided in the Supplementary Information.
